# Insulin Resistance in Type 1 Diabetes: Pathophysiological, Clinical, and Therapeutic Relevance

**DOI:** 10.1210/endrev/bnae032

**Published:** 2025-02-25

**Authors:** Maria Apostolopoulou, Vaia Lambadiari, Michael Roden, George D Dimitriadis

**Affiliations:** Department of Endocrinology and Diabetology, Medical Faculty and University Hospital Düsseldorf, Heinrich-Heine University, 40225 Düsseldorf, Germany; Institute for Clinical Diabetology, German Diabetes Center, Leibnitz Center for Diabetes Research at Heinrich-Heine University, 40225 Düsseldorf, Germany; German Center of Diabetes Research (DZD), Partner Düsseldorf, 85764 München-Neuherberg, Germany; 2nd Department of Internal Medicine, Research Institute and Diabetes Center, National and Kapodistrian University of Athens Medical School, 12462 Athens, Greece; Department of Endocrinology and Diabetology, Medical Faculty and University Hospital Düsseldorf, Heinrich-Heine University, 40225 Düsseldorf, Germany; Institute for Clinical Diabetology, German Diabetes Center, Leibnitz Center for Diabetes Research at Heinrich-Heine University, 40225 Düsseldorf, Germany; German Center of Diabetes Research (DZD), Partner Düsseldorf, 85764 München-Neuherberg, Germany; 2nd Department of Internal Medicine, Research Institute and Diabetes Center, National and Kapodistrian University of Athens Medical School, 12462 Athens, Greece

**Keywords:** type 1 diabetes, insulin resistance, glucotoxicity/lipotoxicity, vascular dysfunction, diabetes therapy

## Abstract

People with type 1 diabetes (T1D) are usually considered to exclusively exhibit β-cell failure, but they frequently also feature insulin resistance. This review discusses the mechanisms, clinical features, and therapeutic relevance of insulin resistance by focusing mainly on human studies using gold-standard techniques (euglycemic–hyperinsulinemic clamp). In T1D, tissue-specific insulin resistance can develop early and sustain throughout disease progression. The underlying pathophysiology is complex, involving both metabolic- and autoimmune-related factors operating synergistically. Insulin treatment may play an important pathogenic role in predisposing individuals with T1D to insulin resistance. However, the established lifestyle-related risk factors and peripheral insulin administration inducing glucolipotoxicity, hyperinsulinemia, hyperglucagonemia, inflammation, mitochondrial abnormalities, and oxidative stress cannot always fully explain insulin resistance in T1D, suggesting a phenotype distinct from type 2 diabetes. The mutual interaction between insulin resistance and impaired endothelial function further contributes to diabetes-related complications. Insulin resistance should therefore be considered a treatment target in T1D. Aside from lifestyle modifications, continuous subcutaneous insulin infusion can ameliorate insulin resistance and hyperinsulinemia, thereby improving glucose toxicity compared with multiple injection insulin treatment. Among other concepts, metformin, pioglitazone, incretin-based drugs such as GLP-1 receptor agonists, sodium-glucose cotransporter inhibitors, and pramlintide can improve insulin resistance, either directly or indirectly. However, considering the current issues of high cost, side effects, limited efficacy, and their off-label status, these agents in people with T1D are not widely used in routine clinical care at present.

## Graphical Abstract

**Graphical Abstract:**
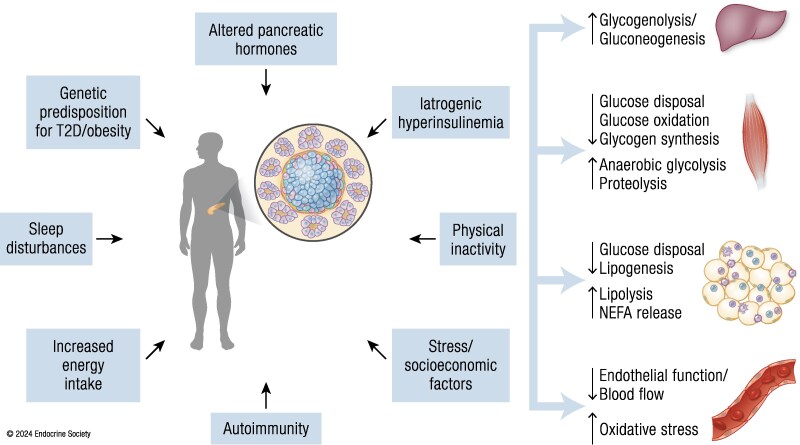

**ESSENTIAL POINTS**

- The common concept that type 1 diabetes only features insulin deficiency, but not insulin resistance, does not hold true, which is of particular relevance in the face of recent therapeutic developments and emerging precision diabetology
- Type 1 diabetes is indeed a heterogeneous disease, as reflected by the identification of different subtypes. In addition to autoimmunity, insulin resistance may play an important role in β-cell destruction and accelerate or even initiate autoimmunity
- Tissue-specific insulin resistance may be detected early during type 1 diabetes progression and aggravate over time. The underlying mechanisms operate synergistically and mutual interaction between insulin resistance and impaired endothelial function can contribute to diabetes-related complications
- Classical lifestyle-related risk factors and peripheral insulin administration induce gluco-/lipotoxicity, hyperinsulinemia, mitochondrial abnormalities, oxidative stress, and inflammation but cannot fully explain insulin resistance in type 1 diabetes, suggesting that other factors are also involved
- Thus, insulin resistance should be considered as an additional treatment target for type 1 diabetes. Lifestyle modifications and continuous subcutaneous insulin infusion can ameliorate insulin resistance and hyperinsulinemia compared with intensified multiple injection insulin treatment. Other concepts have been tried along with insulin, such as metformin, pioglitazone, incretin-based drugs such as GLP-1RAs and novel coagonists, sodium-glucose cotransporter inhibitors, and pramlintide. However, considering the current issues of high cost, side effects, limited efficacy, and their off-label status, widespread use of these agents in people with type 1 diabetes cannot be recommended at present but may play a role in future treatment concepts once outcome trials are available

Insulin resistance is a reversible evolutionary preserved mechanism to ensure survival (1). Chronic insulin resistance along with a compensatory increase in insulin release is a hallmark of obesity, type 2 diabetes (T2D), and metabolic dysfunction–associated steatotic liver disease (MASLD) (1). The presence of insulin resistance in type 1 diabetes (T1D) has long been neglected and any insulin resistance in T1D has been mostly accounted for by glucose toxicity due to chronic hyperglycemia (2). Nevertheless, the underlying pathophysiology of insulin resistance in T1D may involve additional factors such as lipotoxicity, low-grade inflammation, peripheral hyperinsulinemia, and hyperglucagonemia (2, 3). Although T1D has been characterized primarily by autoimmune destruction of β-cells, its pathophysiology seems to be more complex (4). Indeed, it has been even hypothesized that the generation of autoantibodies in T1D may be a secondary event triggered by environment- and lifestyle-related processes leading to islet destruction, and that distinct pathogenetic mechanisms are driving β-cell loss during disease progression (5). Insulin resistance could play a pathogenetic role in this process by forcing compensatory insulin secretion to maintain normoglycemia and thus promote antigen presentation and faster loss of β-cell functionality (6). Accordingly, T1D onset in genetically susceptible individuals seems to occur more often under insulin-resistant conditions such as puberty and infections (7). Moreover, insulin resistance, a well-established factor for vascular complications in T2D, is also associated with cardiovascular events and all-cause mortality in T1D (8, 9).

This review aims to integrate current evidence on the features and natural course, clinical significance, and therapeutic options of insulin resistance in T1D. We have focused mainly on studies investigating insulin resistance by the gold-standard technique, the glucose–insulin clamp, which allows comparison of individuals with T1D with glucose-tolerant humans at identical glycemia and insulinemia.

The relevant literature was retrieved by searching for the terms insulin resistance, autoimmunity, hepatic/muscle/adipose tissue/whole-body insulin resistance, glucagon, islet amyloid polypeptide (IAPP), mitochondria/oxidative stress, metabolic memory/legacy effect, sleep, insulin treatment, metformin, SGLT-2 inhibitors, GLP-1 receptor agonists, exercise, physical activity/inactivity, glucotoxicity, lipotoxicity, MASLD/metabolic dysfunction–associated steatohepatitis, and obesity, all related to T1D from 1980 to June 2024 in PubMed. Further references were identified by analyzing the retrieved publications and by the authors’ files.

## Contribution of Individual Tissues to Insulin Resistance

Insulin plays a central role in metabolic homeostasis by favoring glucose and lipid storage, and protein synthesis. Specifically, insulin regulates both blood glucose concentrations via inhibiting endogenous glucose production (EGP) (mainly by the liver), and glucose disposal (mainly by skeletal muscle and adipose tissue), as well as circulating nonesterified fatty acids (NEFAs) by inhibiting lipolysis (mainly in adipose tissue) and triglyceride synthesis in liver and other tissues (10, 11). Insulin resistance generally results from the combined impairment of insulin sensitivity (ie, the shift of the insulin concentration–effect curve to higher insulin concentrations), and of insulin responsiveness (ie, the reduction of the maximal effect of insulin in its target tissues) (1, 10).

In Table 1, the contribution of liver, skeletal muscle, adipose tissue, and blood flow in insulin resistance is presented along with possible etiological factors in individuals with T1D investigated with euglycemic–hyperinsulinemic clamps (12-56).

**Table 1.** Demographic characteristics, glycemic control, insulin dose per day, and resistance of glucose disposal (GD), blood flow (BF), endogenous glucose production (EGP), and lipolysis to insulin in individuals with T1D investigated with euglycemic–hyperinsulinemic clamps

	Age (years)	Diabetes duration (years)	Overweight/obesity BMI (kg/m^2^) or % IBW*^a^*	Hyperglycemia		Insulin dose (units/day)	Steady-state levels during clamp		Insulin resistance (GD/BF stimulation and EGP/lipolysis suppression by insulin vs controls)			
				HbA1c (%)	FPG (mg/dL)		Glucose (mg/dL)	Free insulin (mU/L)	GD	EGP	Lipolysis	BF (muscle)
DeFronzo et al (12)	33.0 ± 2	14.0 ± 2	101 ± 2 %	NR	165 ± 4	38.0 ± 4	90.0 ± 1	107 ± 3	48%	95%	NR	NR
Lager et al (13)	32.0 ± 4	17.0 ±5	22.0 ± 3	9.70 ± 0.6	164 ± 11	48.0 ± 4	∼90	70.0 ± 9	52%	NR	NR	NR
Del Prato et al (14)	38.0 ± 5	18.0 ± 4	95.0 ± 3%	10.9 ± 0.6	262 ± 32	62.0 ± 12	126 ± 8	117 ± 6	36%	99%	NR	NR
Yki-Järvinen et al (15)	25.0 ± 1.1	7.90 ± 1	102.4 ± 2.7 %	10.5 ± 0.4	153 ± 14	47.0 ± 4	88.0 ± 2	91.0 ± 4	65%	95%	NR	NR
Pernet et al (16)	34.0 ± 3	19.0 ± 4	108 ± 4 %	9.80 ± 0.5	NR	43.0 ± 5	88.0 ± 0.5	21.0 ± 5	42%	NR	NR	NR
							>>	82.0 ± 14	67%	NR	NR	NR
							>>	145 ± 24	85%	NR	NR	NR
							>>	565 ± 120	100%	NR	NR	NR
Yki-Jarvinen et al (17)	34.0 ± 4	10.0 ± 3	114 ± 4 %	11.0 ± 0.6	160 ± 11	46.0 ± 5	86.0 ± 2	81.0 ± 5	57%	NR	NR	NR
Simonson et al (18)	33.0 ± 3	16.0 ± 2	95.0 ± 3 %	11.2 ± 0.6	140 ± 17	42.0 ± 3	88.0 ± 1	90.0 ± 12	56%	98%	NR	NR
Kruszynska et al (19)	36.0 ± 5	13.0 ± 3	25.0 ± 1	11.0 ± 0.5	NR	54.0 ± 6	72.0 ± 0.8	86.0 ± 1.8	60%	NR	NR	NR
Tessari et al (20)	32.0 ± 4	17.0 ± 2	23.0 ± 0.6	7.30 ± 1	NR	57.0 ± 8	90.0 ± 3	36.0 ± 4	33%	NR	NR	NR
							>>	80.0 ± 13	61%	NR	NR	NR
							>>	1249 ± 107	81%	NR	NR	NR
Keller et al (21)	50.0 ± 5	17.0 ± 4	103 ± 5 %	7.00 ± 0.5	140 ± 11	76.0 ± 3	140 ± 11	42.0 ± 4	67%	100%	100%	NR
Trevisan et al (22)	32.0 ± 4	16.0 ± 2	100 ± 3.1 %	7.03 ± 0.7	156 ± 17	55.0 ± 7	87.0 ± 4	36.0 ± 2	47%	100%	53%	NR
							85.0 ± 17	80.0 ± 5	58%	100%	70%	NR
							85.0 ± 3	1249 ± 44	77%	100%	85%	NR
Yki-Järvinen et al (23)	34.0 ± 4	8.00 ± 2	113 ± 5 %	10.6 ± 0.7	NR	42.0 ± 0.2	86.0 ± 2	70.0 ± 6	63%	NR	NR	NR
Hother-Nielsen et al (24)	22.0 ± 0.6	6.40 ± 0.6	21.0 ± 0.2	NR	95.0 ± 0.07	45.0 ± 2	99.0 ± 2	18 ± 0.7	34%	59%	NR	NR
							>>	28 ± 1.1	52%	87%	NR	NR
Yki-Järvinen et al (25)	29.0 ± 3	13.0 ± 2	23.9 ± 0.6	10.6 ± 0.4	245 ± 25	55.0 ± 3	111 ± 7	35.0 ± 3	55%	100%	NR	100%
							92.0 ± 1	132 ± 9	66%	100%	NR	100%
Baron et al (26)	37.0 ± 3	11.4 ± 2	22.0 ± 1	14.0 ± 1	209 ± 36	NR	70.0 ± 2	560 ± 75	67%	NR	NR	65%
Yki-Jarvinen et al (27)	29.0 ± 2	10.0 ± 2	23.0 ± 1	8.20 ± 0.5	130 ± 7	43.0 ± 4	101 ± 2	121 ± 7	79%	NR	NR	NR
Nuutila et al (28)	28.0 ± 2	5.0 ± 1	23.0 ± 1	8.50 ± 1.8	157 ± 23	46.0 ± 3	83.0 ± 2	88.0 ± 3	69%	NR	100%	NR
Mäkimattila et al (29)	35.0 ± 2	19.0 ± 2	25.0 ± 0.7	8.60 ± 0.2	151 ± 13	49.0 ± 3	97.0 ± 2	58.0 ± 3	57%	NR	NR	100%
Makimattila et al (30)	33.0 ± 3	1.07 ± 3	24.0 ± 0.6	8.30 ± 0.1	NR	63.0 ± 4	94.0 ± 2	395 ± 20	70%	NR	NR	100%
Cline et al (31)	34.0 ± 5	NR	24.2 ± 0.8	13.6 ±1.4	NR	NR	162 ± 2	67.0 ± 3	60%	NR	NR	NR
Westerbacka et al (32)	28.0 ± 2	18.0 ± 3	23.9 ± 1	7.60 ± 0.3	133 ± 14	NR	95.0 ± 2	63.0 ± 5	56%	NR	NR	100%
							92.0 ± 2	139 ± 9	63%	NR	NR	100%
Raitakari et al (33)	30.0 ± 0.4	11.0 ± 2	24.5 0.4	9.80 ± 0.98	117 ± 9	60.0 ± 7	104 ± 3	333 ± 30	72%	NR	NR	50%
Ekstrand et al (34)	27.0 ± 0.3	12.7 ± 0.6	72.0 ± 2 kg*^b^*	8.60 ± 0.3	NR	NR	103 ±1	69.0 ± 6	60%	NR	NR	NR
Ducluzeau et al (35)	33.0 ± 3	16.0 ± 3	24.0 ± 1	9.20 ± 0.3	227 ± 14	45.0 ± 5	101 ± 4	158 ± 19	83%	NR	NR	NR
Peltoniemi et al (36)	23.0 ± 1	7.0 ± 1	23.0 ± 0.4	7.10 ± 0.4	178 ± 25	56.0 ± 5	97.0 ± 2	55.0 ± 4	56%	NR	NR	100%
Greenfield et al (37)	39.0 ± 7	23.8 ± 3	24.7 ± 1	8.10 ± 0.3	169 ± 1	45.0 ± 3	∼90	47.0 ± 4	67%	NR	NR	NR
Perseghin et al (38)	31.0 ± 2	20.0 ± 2	22.5 ± 0.7	8.60 ± 0.4	229 ± 25	52.0 ± 3	∼90	70.0 ± 4	70%	78%	91%	NR
Perseghin et al (39)	35.0 ± 2	21.0 ± 1	22.7 ± 0.6	8.70 ± 0.3	200 ± 5	52.0 ± 3	117 ± 18	64.0 ± 3	72%	76%	NR	NR
Rosenfalck et al (40)	35.0 ± 3	9.60 ± 3	25.1 ± 2	8.50 ± 0.3	NR	50.0 ± 5	∼90	∼80	52%	NR	100%	NR
Nadeau et al (41)	15.60 ± 0.7	7.50 ± 1	20.9 ± 0.9	8.70 ± 0.5	132 ± 13	NR	∼90	∼200	63%	NR	46%	71%
Maahs et al (42)	46.0 ± 1.5	23.0 ± 1.3	27.0 ± 0.7	7.50 ± 0.1	117 ± 6	NR	89.0 ± 0.5	106 ± 5	45%	NR	NR	NR
Kacerofsky et al (43)	36.0 ± 2.8	17.0 ± 5	26.0 ± 1	6.80 ± 0.1	115 ± 17	NR	99.0 ± 1	99.0 ± 6	50%	NR	100%	NR
Hsu et al (44)	25.4 ± 1	6.10 ± 1.3	23.4 ± 0.5	6.90 ± 0.35	NR	NR	∼90	∼200	88%	NR	100%	NR
Bravard et al (45)	29.0 ± 1.5	NR	24.5 ± 1.3	9.10 ± 0.4	243 ± 16	45.0 ± 5	101 ± 4	158 ± 19	91%	NR	NR	NR
Schauer et al (46)	45.0 ± 2	23.0 ± 1	27.0 ± 0.7	7.50 ± 0.14	116 ± 6	NR	89.0 ± 0.5	106 ± 6	44%	NR	50%	NR
Pereira et al (47)	46.0 ± 2	22.5 ± 1	27.1 ± 0.7	7.50 ± 0.1	116 ± 6	NR	∼90	∼100	45%	NR	NR	NR
Shay et al (48)	48.0 ± 1	39.0 ± 1	26.9 ± 0.9	7.60 ± 0.2	NR	NR	∼90	∼100	71%	NR	NR	NR
Bergman et al (49)	45.0 ± 2	22.9 ± 1.7	26.6 ± 0.9	7.70 ± 0.16	NR	NR	∼80	∼100	38%	41%	NR	NR
Rathsman et al (50)	18.0 ± 0.3	7.30 ± 0.9	23.5 ± 0.85	8.90 ± 3.9	155 ± 14	64.0 ± 3	∼90	∼100	70%	NR	NR	NR
Donga et al (51)	45.0 ± 16	26.0 ± 4	23.5 ± 2.2	7.40 ± 2.5	NR	35.0 ± 9	90.0 ± 2	24.0 ± 2	61%	81%	61%	NR
Cree-Green et al (52)	16*^c^*	NR	24*^c^*	8.3*^c^*	NR	NR	∼98	∼200	61%	80%	70%	NR
Williams et al (53)	48.0 ± 1.5	39.0 ± 2	26.2 ± 0.9	7.60 ± 0.3	NR	44.0 ± 18	∼90	∼100	70%	NR	NR	NR
Jahn et al (54)	29.0 ± 4	17.0 ± 3	25.0 ± 1	7.40 ± 0.3	168 ± 22	NR	∼130	∼65	76%	NR	NR	13%*^d^*

The percentage of GD/BF stimulation and EGP/Lipolysis suppression by insulin in T1D has been compared to that of age/BMI-matched healthy individuals considered as 100%.

Abbreviations: BF, blood flow; EGP, endogenous glucose production; FPG, fasting plasma glucose; GD, glucose disposal; IBW, ideal body weight.

^*a*^Analysis of BW according to IBW (Metropolitan Life Insurance Tables): obese >120% IBW; overweight 110-120% IBW; adequate 90-109% IBW; underweight 80% to 89% IBW. The results of BF refer to macrovasculature.

^*b*^Only body weight in kg is reported.

^*c*^The results are expressed as mean ± SEM except those reported as median.

^*d*^The results of BF refer to macrovasculature except those refering to microvasculature.

**Figure 1.**
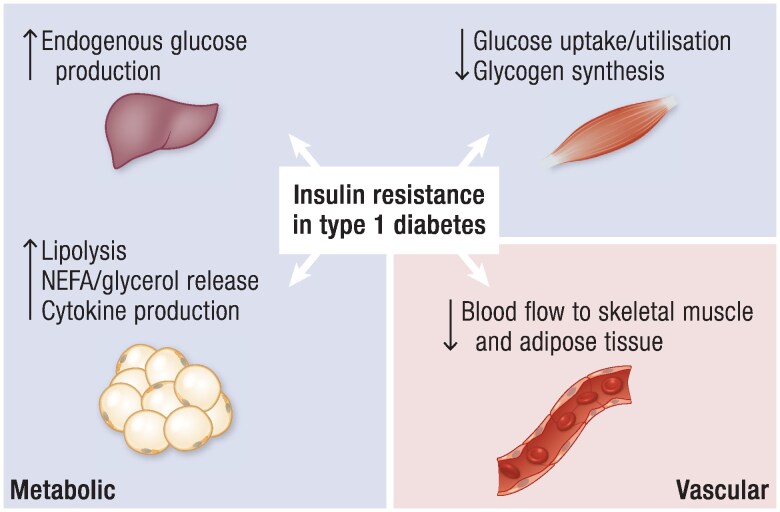
In T1D, studies with euglycemic–hyperinsulinemic clamps showed insulin resistance in the liver, skeletal muscle, adipose tissue, and vascular endothelium leading to an increase in endogenous glucose production, a decrease in glucose transport/utilization and glycogen synthesis, an increase in lipolysis and release of NEFA/pro-inflammatory cytokines, and a decrease in blood flow, respectively. NEFA, nonesterified fatty acids).

The euglycemic–hyperinsulinemic clamp test is the gold-standard technique to measure insulin sensitivity and responsiveness directly by infusing insulin to achieve defined plasma concentrations, and glucose to maintain glycemia at fasting or postprandial levels. Combination of the clamp technique with intravenous infusions of labeled metabolites, indirect calorimetry, and/or multinuclear magnetic resonance spectroscopy (MRS) allows accurate assessment of insulin effects on glucose disposal (M value, Rd), EGP (Ra), and oxidative/nonoxidative glucose and energy metabolism (57). Intravenous glucose tolerance test and oral glucose tolerance test (OGTT) modeling have also been described but should not be used in T1D. Simpler indexes have been developed to facilitate their use in clinical practice. Homeostatic Model Assessment for Insulin Resistance (HOMA-IR), a widely used index, is calculated from fasting glucose and endogenous insulin concentrations and, therefore, reflects insulin sensitivity in the liver rather than muscle (57). Even though this index can be applied before T1D onset, it does not apply following diagnosis and insulin treatment due to the absence of endogenous insulin secretion. The estimated glucose disposal rate (eGDR) has been developed as a surrogate marker for insulin resistance in T1D, calculated from waist circumference, blood pressure, and HbA1c (58). However, eGDR should be interpreted with caution because it relies on factors known to relate directly to insulin resistance and thereby does not allow independent assessment of insulin sensitivity or responsiveness. The eGDR originated from examination of 24 individuals with inadequate glycemic control (average HbA1c of 9.9%) (58), which by itself could have affected insulin resistance. Also, the included parameter HbA1c can be confounded by anemia, hemoglobinopathies, or chronic kidney disease. Furthermore, the parameter blood pressure can be affected by specific causes of arterial hypertension or antihypertensive agents given for other indications. Hypertension may also develop at later stages and therefore underestimate the eGDR in younger individuals with T1D (59). Of note, certain components of eGDR are not always associated with clamp-derived measures of insulin resistance (see below). In the following subsections, target tissues involved in metabolic and vascular insulin resistance in T1D are presented (Fig. 1).

### Hepatic Insulin Resistance

The liver plays a key role in glucose homeostasis by storing glucose as glycogen via the direct and indirect/gluconeogenic pathway in the postprandial state, and by releasing glucose into the circulation via glycogenolysis and gluconeogenesis in the fasting state to maintain normoglycemia (10, 11, 60) (Table 1). This fine-tuning of glycemia in healthy individuals is mainly controlled by the portal venous insulin to glucagon ratio, which is increased postprandially favoring the incorporation of glucose into glycogen and decreased during fasting to allow for glycogen breakdown and gluconeogenesis (61-63). In addition, glycemia per se contributes to hepatic glucose metabolism by regulating glycogen turnover (62, 64).

In individuals with T1D, fasting EGP before clamp-induced hyperinsulinemia was either higher or similar to glucose-tolerant participants even in the presence of hyperglycemia. However, in the latter case, the rather low rates of EGP may still indicate relative overproduction of glucose (18), as even moderate hyperglycemia should suppress EGP by more than 90% independently of insulin (65). Therefore, high fasting glucose levels should have reduced EGP in the individuals with T1D (66). Elevations of fasting plasma glucagon, lactate, NEFAs, glycerol, and amino acids in T1D may explain, at least in part, the increase in fasting rates of EGP: NEFAs and glucagon are potent stimulators of gluconeogenesis, whereas lactate, glycerol, and amino acids are important substrates for gluconeogenesis (15, 18, 22, 38, 39, 52, 60, 67).

As to insulin-mediated regulation of EGP, some studies reported only partial EGP suppression during both physiological (24, 38, 39, 49, 51) and supraphysiological hyperinsulinemia (52) suggesting hepatic insulin resistance, whereas other studies found no differences compared with glucose-tolerant people, despite comparable insulinemia (12, 14, 15, 18, 21, 22, 55, Table 1). Since the liver is more sensitive to insulin than skeletal muscle (68, 69), the degree of insulinemia during clamps in some studies might have been too high to detect small differences in hepatic insulin sensitivity (24, 51). Another technical explanation for the observed discrepancies may be the use of overnight insulin administration to achieve euglycemia in hyperglycemic persons with T1D, which could have already decreased fasting EGP and modified the subsequent responses to insulin during the clamps (19, 24, 30, 52, 56).

Hepatic EGP is mainly determined by portal venous insulin levels so the assessment of insulin-mediated EGP suppression should be investigated in terms of calculated portal rather than peripheral (systemic) insulin concentrations (70). Of note, portal insulin levels are 2- to 3-fold higher than in the peripheral circulation under physiological conditions (71). Consequently, in the absence of endogenous insulin secretion, intravenous insulin infusions produce nearly identical or even higher insulin levels in the peripheral compared with portal circulation (49, 72). During hyperinsulinemic clamp tests, people with T1D will therefore develop a markedly higher peripheral to portal vein insulin gradient than glucose-tolerant humans (24, 49). In T1D, in contrast to the rightward shift in the dose–response curves for EGP as a function of peripheral insulin levels, these curves have been found leftward shifted when expressed as a function of portal insulin concentrations compared with healthy individuals; this was due to the rightward shift of the control curve when expressed as a function of calculated portal insulin levels which were higher than peripheral (24).

In summary, the euglycemic–hyperinsulinemic clamp studies revealed that individuals with T1D show insulin resistance when EGP evaluation was based on peripheral insulinemia, but not when based on portal venous insulinemia (24, 49, 73).

### Skeletal Muscle Insulin Resistance

Under euglycemic– or hyperglycemic–hyperinsulinemic clamp conditions, more than 80% of the infused glucose is taken up by skeletal muscle (68) (Table 1). Thus, skeletal muscle is the principal site of insulin-stimulated glucose disposal and thereby also primarily responsible for whole-body insulin resistance (1, 2, 60).

In contrast to the inconsistent findings for hepatic insulin resistance in T1D, most studies investigating insulin-sensitive whole-body glucose disposal revealed insulin resistance. During physiological postprandial insulinemia (69), whole-body glucose disposal was about 40% lower in individuals with T1D than in age/body mass index (BMI)–matched healthy glucose-tolerant people (12-25, 28, 29, 31-34, 36-40, 42, 43, 46-51, 53-56). At hyperinsulinemia exceeding postprandial levels (69), glucose disposal was found either lower (20, 22, 25-27, 30, 32, 35, 44, 52) or similar to healthy humans (16, 45, 56), suggesting decreased or normal responsiveness to insulin, respectively. The reduction in overall insulin-mediated glucose disposal in T1D may be due to lower glucose uptake and storage in skeletal muscle. This is supported by findings that the insulin-mediated glucose disposal at the level of the whole body (40%) is similar to that in the forearm (41%) or femoral muscles (47%) in people with T1D (25, 28-30, 32, 36). Since glucose can regulate its uptake and metabolism in muscle independently of insulin (74), the presence of hyperglycemia in T1D may provide a compensatory mechanism to maintain a relatively “normal” glucose disposal in the presence of insulin deficiency or insulin resistance (12).

Following the entry into muscle cells by the GLUT-4 transporters, glucose is metabolized via oxidative and nonoxidative pathways. Nonoxidative glucose disposal occurs by the storage of glucose as glycogen and by nonoxidative glycolysis leading to lactate formation (1, 2). In muscle, glycogen synthesis is the major nonoxidative pathway for glucose metabolism and is closely associated with the rates of glucose disposal in this tissue (75). In insulin-resistant people with T1D, under conditions of euglycemic- or hyperglycemic-hyperinsulinemia, the reduction in whole-body and forearm/femoral glucose disposal was correlated positively with similar reductions in nonoxidative glucose metabolism, glycogen synthesis, and glycogen synthase activity, whereas lactate formation was found either lower or similar to controls; glucose oxidation and pyruvate dehydrogenase activity were unchanged (19, 22, 28, 31, 34, 49). The intramuscular contents of free glucose, glucose-6-phosphate, and glycolytic intermediates were not accumulated during insulin stimulation suggesting abnormal glucose transport rather than other steps in intracellular glucose metabolism; this has been supported by impaired regulation of muscle GLUT-4 gene expression by insulin (25, 27, 35). These findings were validated by using noninvasive ^1^H/^31^P MRS to monitor glucose and ATP fluxes in vivo in overweight individuals with T1D (43). The marked muscle insulin resistance was characterized by lower insulin-stimulated myocellular glucose 6-phosphate and ATP synthesis indicating impaired muscle glucose transport/phosphorylation, which was positively associated with HbA1c but not with plasma NEFA levels or intramyocellular lipid accumulation.

To summarize, insulin resistance of muscle glucose disposal is a consistent finding in T1D. Measurements of free glucose, glycolytic intermediates, and enzymes suggest that the reduction in insulin-mediated glucose disposal is due to lower transport of glucose and its subsequent storage as glycogen.

### Adipose Tissue Insulin Resistance

Basal plasma NEFA and glycerol levels in T1D were found to be either normal (21, 22, 28, 40, 43, 46, 51, 52), increased (38, 39), or decreased (43) (Table 1). At physiological postprandial insulinemia, the suppression of lipolysis was impaired by 50% to 70% in individuals with T1D than in age/BMI-matched healthy glucose-tolerant people indicating insulin resistance (22, 46, 51). On the contrary, other investigators reported normal suppression of lipolysis at similar insulin levels within the physiological postprandial range (21, 28, 38, 40, 43). Lipolysis is highly sensitive to insulin (70) so that differences between studies could at least partly result from overnight insulin administration to reduce hyperglycemia before beginning the clamps or from the high clamp insulinemia, which leads to complete suppression of lipolysis in T1D (22, 44). Several adipokines are important modulators of insulin sensitivity and are dysregulated in T2D (76). In T1D, adiponectin levels were reported to be about 30% higher than in healthy humans and associated positively with age and diabetes duration, but inversely with weight-adjusted daily insulin dose, BMI, and percentage trunk and visceral adipose tissue (VAT) volume (38, 39, 41, 44, 46, 47, 52). Adiponectin positively related to insulin-stimulated glucose disposal in both humans with and without T1D. In people with T1D, the correlation showed a shift to the right, indicating lower glucose disposal rates at a given adiponectin level, and thereby a higher degree of insulin resistance (47). These reports suggest that adiponectin does not seem to play a causal role in insulin resistance of T1D. Moreover, people with T1D do not show alterations in plasma levels of leptin or RBP-4 compared with age/BMI-matched healthy glucose-tolerant people (44, 52).

To summarize, impaired insulin-mediated suppression of plasma glycerol and NEFA levels during euglycemic–hyperinsulinemic clamps indicated higher rates of lipolysis and suggested adipose tissue insulin resistance in T1D.

### Vascular Insulin Resistance

In addition to cellular alterations of glucose extraction, vascular abnormalities may also be responsible for muscle insulin resistance in T1D (Table 1). Insulin affects both macrovascular and microvascular endothelium and increases muscle and adipose tissue blood flow by vasodilation and capillary recruitment mediated by NO-dependent processes (77, 78). The effects of insulin on blood flow and tissue glucose disposal and metabolism are tightly coupled processes and, therefore, important determinants of tissue sensitivity to insulin (79-81).

Muscle blood flow enhancement in response to insulin could be due to increased flow velocity in the macrovasculature, increased capillary recruitment and tissue blood volume in the microvasculature, or both (77, 78). In T1D, blood flow responses to insulin have been examined to address the question of whether reduced insulin-stimulated glucose disposal could be attributed to a decrease in skeletal muscle blood flow and capillary exchange of glucose or to intrinsic defects in myocellular glucose transport/metabolism.

In individuals with T1D without complications, the stimulation of total blood flow velocity in the forearm and femoral muscles by physiological postprandial hyperinsulinemia was unchanged, while insulin-stimulated glucose uptake and arteriovenous glucose differences across muscle groups were 40% lower in participants with T1D vs healthy humans, suggesting that a defect in glucose extraction rather than blood flow was responsible for insulin resistance. Arteriovenous glucose differences and glucose disposal but not blood flow rates were positively associated, suggesting that although blood flow and glucose disposal are coupled processes the latter may be impaired even when blood flow and glucose delivery are normal (25, 29, 32, 36). On the contrary, at hyperinsulinemia much higher than postprandial levels, blood flow rather than glucose extraction might be rate-limiting for glucose disposal (26, 33, 41).

Insulin may directly enhance perfusion in muscle microvasculature changing flow distribution and increasing glucose delivery even without altering total limb blood flow (78, 80). Therefore, assessing the association between vascular and metabolic actions of insulin in muscle requires more than examining only total limb blood flow. In participants with T1D, insulin-stimulated glucose disposal, and blood volume (capillary recruitment) in the forearm microvasculature were both severely impaired and positively associated with each other, independent of HbA1c, BMI, and diabetes duration (54, 82).

During euglycemic–hyperinsulinemia, blood flow has been assessed with various techniques such as strain-gauge plethysmography, thermodilution, or positron emission tomography combined with intravenous administration of [^15^O]H_2_O (25, 26, 29, 32, 33, 36, 41). Although these techniques may offer similar sensitivity in detecting changes in blood flow during insulin infusion (77, 83), the clinical studies revealed a broad range of results. This is therefore likely attributable to the study design or cohort selection, such as the dose/duration of insulin infusion during clamps (longer duration and higher doses enhance blood flow rates), limb muscularity, physical fitness, and T1D duration/poor glycemic control possibly inducing endothelial damage (83). Of note, even lean, glucose-tolerant, and insulin-sensitive individuals exhibit a large interindividual variation of the vasodilatory action of insulin with an effective dose for maximal vasodilation ranging from an average of 40 to more than 100 mU/L (77).

To summarize, reduction of insulin-stimulated blood flow indicates vascular insulin resistance in T1D in addition to metabolic insulin resistance and this may contribute to abnormal insulin-stimulated glucose disposal in T1D.

## The Natural Course of Insulin Resistance in T1D

There is evidence that, along with the autoimmune attack, insulin resistance is present in T1D, supporting the concept that changes in insulin sensitivity may play a role in the progression of this disease (7).

### Ιnsulin Resistance Before Clinical Diagnosis of T1D

The time until clinical diagnosis of T1D may span years in autoantibody-positive individuals and the rate of progression can vary by several modifiers including insulin resistance (84). The “accelerator hypothesis” postulated that T1D and T2D represent 2 sides of 1 coin. The differentiation lies in the more intense and rapid destruction of β-cells in T1D affected by 3 accelerators: higher rates of genetically determined apoptosis, development of insulin resistance, and aggressive autoimmune reaction (85-87). Of these accelerators, insulin resistance, mostly but not exclusively due to obesity, is of major clinical importance as a modifiable risk factor and therefore a target for potential disease prevention (84, 85).

First- and second-degree relatives of family members with T1D who were euglycemic and nonobese despite islet autoantibody positivity were followed for 4 years until disease onset. Those who progressed more rapidly to T1D featured 10% and 30% increased fasting glucose and insulin levels, respectively. However, in these people, first-phase insulin secretion was almost completely lost, HOMA-IR was elevated by 50%, inversely correlated with the time of T1D onset, and was the only risk factor independently associated with rapid progression to overt T1D vs nonprogressors (88, 89).

A similar cohort of first- and second-degree relatives of people with T1D underwent assessments of the contribution of insulin resistance (mathematical modeling of glucose/C-peptide responses after OGTT) and β-cell glucose sensitivity (slope of the insulin secretion/plasma glucose dose–response function) every 6 months for 3 years until the onset of hyperglycemia and disease progression (90). These high-risk individuals exhibited abnormality of β-cells to respond to changes in plasma glucose levels followed by increases in postprandial and fasting hyperglycemia years before diagnosis of T1D. A rapid progression of insulin resistance characterized the transition to excessive hyperglycemia and β-cell insufficiency. Even after diagnosis, autoantibody-positive people initially diagnosed with T2D not requiring insulin treatment exhibited hyperglycemia in the presence of insulin resistance and partly reserved β-cell function (91).

To summarize, insulin resistance may be present already in preclinical stages of T1D, along with low β-cell function and dysglycemia before the clinical onset of overt T1D. Several mechanisms may be responsible for insulin resistance at this stage of progression: increased growth hormone secretion in mid-childhood and during adolescence (92-94), increased glucagon secretion due to intra-islet insulin deficiency (see “Glucagon, Islet Amyloid Polypeptide” and “Evidence for Insulin Resistance Preceding Autoimmunity”), infections (95), and/or obesity (see “Obesity”). However, the participants in these studies (88-91) were already autoantibody positive, and hence potential causality between insulin resistance and autoimmunity cannot be established.

### Ιnsulin Resistance After Clinical Diagnosis of T1D

**Figure 2.**
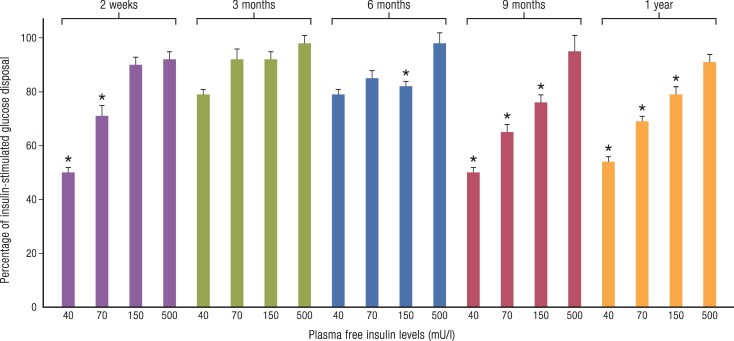
Percentage (%) of insulin-stimulated whole-body glucose disposal during 4-step euglycemic–hyperinsulinemic clamps (average of plasma free insulin levels 40, 70, 150, and 500 mU/L) in people with T1D 2 weeks, 3 months, 6 months, 9 months, and 1 year after diagnosis and initiation of insulin treatment compared with age/BMI-matched healthy controls. At physiological insulin levels, insulin-stimulated glucose disposal was significantly lower even at 2 weeks following diagnosis, was transiently normalized at 3 months (during the so-called “honeymoon” period), started to decline again at 9 months to 1 year, and after that remained at levels 40% lower in all study groups. Insulin resistance was positively associated with HbA1c and insulin dose per day during the study. *P < .01 vs results in age/BMI-matched healthy individuals considered as 100%s (data calculated from reference 56).

In 2 prospective trials (55, 56), clamp-measured insulin sensitivity was stratified according to diabetes duration and glycemic control at 2 weeks, 3 to 6 months, and 9 months to 1 year after diagnosis. Hepatic insulin sensitivity was unchanged in all groups. However, glucose disposal assessed at physiological postprandial insulin levels, decreased already at 2 weeks, transiently normalized at 3 months (during the so-called “honeymoon” period), declined again at 9 months to 1 year, and remained about 40% lower thereafter in all groups than in healthy humans (Fig. 2). Of note, glucose disposal was normally suppressed at insulin levels much higher than the postprandial range. Insulin resistance was positively associated with HbA1c and daily insulin dose during treatment. In the cross-sectional part of these trials (55, 56), all groups with long-standing T1D (2-32 years) showed 40% lower insulin-stimulated glucose disposal 40% than healthy individuals. Albeit within the normal range, BMI was inversely associated with insulin action, suggesting that even a relative increase in body weight within the normal range may be responsible, at least in part, for insulin resistance (55). A more recent study in persons with overt T1D showed inverse associations of eGDR with waist circumference, waist to hip ratio, lipid accumulation product, and body adiposity index (96). The German Diabetes Study (GDS) monitors whole-body insulin sensitivity using euglycemic–hyperinsulinemic clamps in people with recent onset diabetes every 5 years for up to 20 years (97). At times of diagnosis, insulin-stimulated glucose disposal was 30% lower than in age-matched healthy humans and remained lower for the next 5 years (98, 99). Within 12 months after diagnosis, insulin resistance correlated positively with insulin antibody titers even after adjustments for sex, age, BMI, daily insulin dose, and β-cell residual secretory capacity (100). The GDS also showed that the percentage of cytotoxic CD8+ T-cells—known to be mainly responsible for β-cell destruction in T1D (4)—was positively associated with insulin resistance, hyperglycemia, NEFAs, and high-sensitive C-reactive protein at 5 years but not within the first year after diagnosis (101, 102).

To summarize, the presence of insulin resistance persists after diagnosis of T1D, may subside temporarily, and is positively associated with glycemia, NEFAs, body weight, and markers of low-grade inflammation. The possible underlying mechanisms including hyperinsulinemia are described in the following section.

## Mechanisms

The pathophysiological mechanisms contributing to metabolic and vascular insulin resistance in T1D include glucotoxicity, lipotoxicity, low-grade inflammation, inappropriate (iatrogenic) hyperinsulinemia, impaired mitochondrial function, and oxidative stress. These mechanisms work synergistically to impair endothelial function leading to chronic complications (60, 103).

### Hyperglycemia, Glucotoxicity, and Glucose Variability

**Figure 3.**
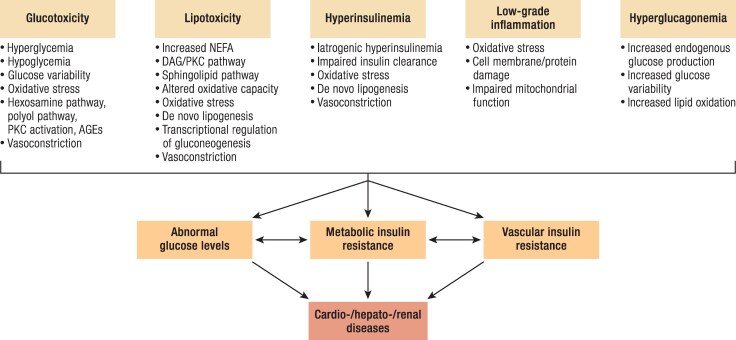
Interacting and shared mechanisms responsible for the development of insulin resistance in T1D involve glucotoxicity (hyperglycemia/hypoglycemia, increased glucose variability, oxidative stress, activation of hexosamine, polyol, and PKC pathways, accumulation of AGEs, and vasoconstriction), lipotoxicity (accumulation of NEFA, activation of DAG/PKC and sphingolipid pathway, altered oxidative capacity, oxidative stress, de novo lipogenesis, transcriptional regulation of gluconeogenesis and vasoconstriction), hyperinsulinemia (iatrogenic hyperinsulinemia, impaired insulin clearance, oxidative stress, de novo lipogenesis, vasoconstriction) low-grade inflammation, and hyperglucagonemia. Mutual interaction between mechanisms related to glucotoxicity, and metabolic/vascular insulin resistance contributes to impaired endothelial function and cardio-/hepato-/renal diseases. Abbreviations: EGP, endogenous glucose production; NEFA, nonesterified fatty acid; DAG diacylglycerol; AGE, advanced glycation end product; PKC, protein kinase-C.

Hyperglycemia has been considered as a main cause of insulin resistance and vascular complications (8, 65, 104) (Fig. 3). Even in people without known diabetes, increases in HbA1c by 1% within the range of 5.5% to 6.5% are associated with an 11% to 16% higher risk for cardiovascular events (105). Both hyperglycemia and insulin resistance share mechanisms also responsible for cardiovascular complications (8,104).

Several studies have linked hyperglycemia with insulin resistance. In T1D, some (25, 29, 30, 36, 38, 43, 55, 56) but not all studies (37, 41, 44, 46, 49, 50, 53) using euglycemic–hyperinsulinemic clamps showed a positive association between HbA1c and insulin resistance (Table 1). However, HbA1c can be a flawed marker of glycemia in some individuals, and this association fails to consider other glycemic markers such as glucose variability (106), or metabolic memory effect (see “Abnormalities Of Mitochondrial Function and Oxidative Stress”) that affect insulin resistance. In the GDS, even in metabolically adequately controlled people with recent-onset T1D (average HbA1c 6.7%), whole-body insulin-stimulated glucose disposal was inversely associated with the degree of fasting glycemia (107), with the sum of plasma levels of hexoses as the strongest predictor for insulin resistance supporting the harmful role of hyperglycemia (108).

In T1D, glucose variability with frequent hyperglycemic peaks and hypoglycemic nadirs due mainly to iatrogenic hyperinsulinemia is an integral component of inadequate metabolic control contributing to the development of oxidative stress, low-grade inflammation, and diabetes-related complications (106, 109). In people with T1D, glucose variability was positively associated with subclinical atherosclerosis (110), and inflammation (111). The latter studies focused on 2 short-term parameters of glucose variability, SD, and coefficient of variation. Of note, data on short-term glucose variability should be interpreted by taking HbA1c into account because previous exposure to excessive hyperglycemia may act as a preconditioning factor and hence minimize the effects of glucose variability on complications and mortality (106, 112). Likewise, hyperglycemia following recovery of hypoglycemia in T1D may lead to increasing oxidative stress, low-grade inflammation, and worsening of endothelial function (113). In autoantibody-positive first-degree relatives of people with T1D and individuals with recent-onset T1D (average HbA1c 8.1%), lower whole-body glucose disposal rate, and higher HOMA-IR values were strongly associated (inversely and positively, respectively) with glucose variability indexes. Interestingly, high glucose variability indexes were as effective as the measures of insulin resistance in predicting first-degree relatives who developed T1D within the 20-month follow-up of the study (114). In a recent report (115) examining the link between insulin resistance and glucometrics (continuous glucose monitoring data) in adults with T1D (median HbA1c 7.3%), no association between insulin resistance estimated by euglycemic–hyperinsulinemic clamps and glucose variability (coefficient of variation) could be observed, whereas there was an association when insulin resistance was measured with eGDR. As discussed by the authors, the lack of significance in the clamp experiments was due to the small number of participants in that group.

In T1D, hyperglycemia also decreases glycocalyx density facilitating the adhesion of inflammatory cells on the endothelial cell layer, thereby leading to activation of the coagulation cascade and atherogenesis (116-118). The glycocalyx serves as a mechanistic sensor of shear stress mediating endothelial NO release to permit insulin-stimulated vasodilation and blood flow increase. Thus, a link between glycocalyx density and insulin resistance seems plausible, but studies in people with T1D are lacking (116, 117). Interestingly, glycocalyx thickness was negatively associated with insulin sensitivity indices (Matsuda index) in glucose-intolerant first-degree relatives of persons with T2D, probably due to hyperglycemia (119).

Hyperglycemia has been considered to be a primary mechanism for muscle and liver insulin resistance (65). Even short-term hyperglycemia (average 360 mg/day for 24 hours) markedly reduces whole-body, forearm, and nonoxidative glucose disposal by 26%, 39%, and 54%, respectively without affecting glucose oxidation. Hyperglycemia reduced muscle glycogen content by 10% without differences in myocellular free glucose, glucose-6-phosphate, and fructose-6-phosphate (43, 120). Indeed, short-term hyperglycemia can decrease the translocation of insulin-stimulated GLUT-4 transporters to the surface membrane of muscle cells and inhibit glucose transport (121), indicating a primary abnormality of glucose transport underlying muscle insulin resistance during hyperglycemia. Combined in vivo ^13^C-MRS/^2^H_2_O with glucose–insulin clamps allowed noninvasive measurements of hepatic glycogen fluxes and gluconeogenesis in nonobese inadequately controlled individuals (average HbA1c 8.8-10.2%) before and after glycemic control. Under postprandial conditions, the rates of net glycogen synthesis were reduced in the presence of a lower portal insulin to glucagon ratio. Although short-term (24 hours) near-normoglycemia by variable intravenous insulin infusion doubled postprandial net glycogen synthesis, only long-term improved glycemic control (HbA1c <7% for over 1 year) by intensified insulin treatment normalized the hepatic net glycogen synthesis rates (122-124). Under fasting conditions, the ∼30% higher EGP due to ∼70% higher rates of gluconeogenesis and ∼70% lower rates of net glycogen synthesis in nonobese inadequately controlled people with T1D (average HbA1c 8.5%, diabetes duration 20 years) were reversed by improved glycemic control (average HbA1c 7%, continuous subcutaneous insulin infusion [CSII]) (125). These studies indicate that hepatic glucose metabolism is not irreversibly altered even in long-standing T1D providing glycemic control is maintained within the range considered as “optimal.”

The molecular mechanisms involve a primary effect of hyperglycemia to enhance mitochondrial production of reactive oxygen species (ROS) (126) which subsequently activates the hexosamine (127) and polyol pathways (128), protein kinase-C isoforms (PKC-β and PKC-δ) (129), and the formation of advanced glycation end-products (130) in capillary endothelial cells. Activation of these pathways further aggravates oxidative stress, increases proinflammatory cytokine production inducing inflammation, and among others increases the expression of endothelin-1, a potent NO antagonist inducing vasoconstriction and ischemia (8, 131). In the GDS cohort, an increase in HbA1c at 2 years after T1D diagnosis was associated with increased levels of interleukin (IL)-18 (132).

Taken together, these reports suggest that hyperglycemia/glucotoxicity and glucose variability are significant factors in the development of insulin resistance in T1D.

### Hyperinsulinemia

**Figure 4.**
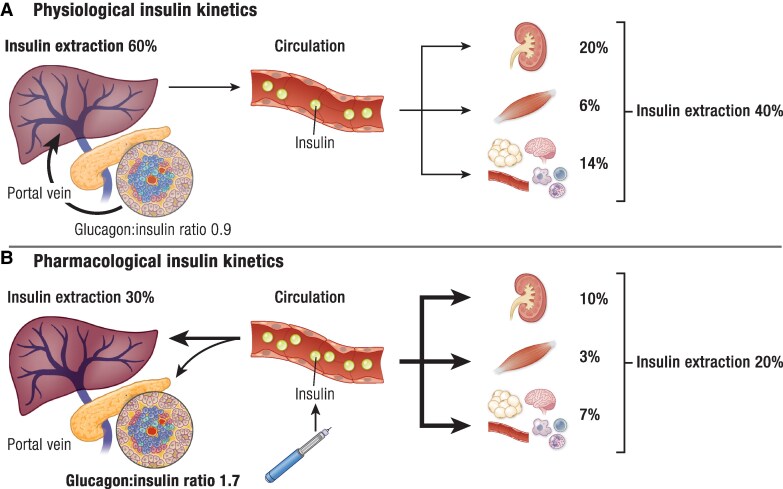
(A) Portal route of insulin following secretion from the pancreatic β-cells. During its first pass through the liver, insulin is degraded by over 60%; the rest enters the systemic circulation to be cleared by the kidneys (20%), skeletal muscle (6%), and other insulin-responsive tissues/cells such as adipose tissue, brain, endothelial cells, and blood cells (14%). The secreted insulin inhibits glucagon release by the α-cells in the islet environment (estimated glucagon/insulin ratio at 0.9). (B) Peripheral route of insulin following subcutaneous administration. In T1D, exogenously administered insulin leads to comparable levels in the peripheral circulation and the portal vein resulting in insufficient suppression of glucagon production (estimated glucagon/insulin ratio at 1.7). Insulin clearance measured by euglycemic–hyperinsulinemic clamps, OGTT, and intravenous glucose tolerance test was found to be about 50% lower compared with healthy individuals (30% in the liver, and 20% in the other insulin-responsive tissues and cells) leading to significant hyperinsulinemia (data from references 39, 133-136).

Following secretion from the β-cells and during its first pass through the liver, insulin is degraded by over 60%; the rest enters the circulation to be cleared by the kidneys (20%), muscle (6%), and the other insulin-sensitive tissues/cells including adipose tissue (14%) (133, 134) (Fig. 3) (Fig. 4). Therefore, under physiological conditions such as after a meal, the liver is the main organ involved in insulin clearance and is exposed to ∼3-fold higher insulin levels than peripheral tissues; this ensures the direct and indirect (through inhibition of adipose tissue, lipolysis, and NEFA release) suppression of EGP by insulin and the increase in muscle glucose disposal, avoiding peripheral hyperinsulinemia and the risk of adverse effects (137, 138).

Overfeeding, especially with excess carbohydrates, can rapidly decrease hepatic insulin clearance by about 15% leading to systemic hyperinsulinemia (139). In people with obesity, insulin clearance is significantly decreased and inversely associated with insulin resistance in the liver, muscle, and adipose tissue; lower insulin clearance may be an important compensatory mechanism that contributes to hyperinsulinemia in these clinical conditions (135). In normoglycemic autoantibody-positive children with a family history of T1D, insulin clearance in the fasting state and after an OGTT was decreased by 36% and 53%, respectively, and was associated positively with impaired β-cell responsiveness and negatively with insulin resistance (140). In the GDS (136) insulin clearance was investigated in adults with recent-onset T1D (average HbA1c 6.5%, BMI 25 kg/m^2^) with euglycemic–hyperinsulinemic clamps and measurements of C-peptide to insulin ratios during oral or intravenous tolerance tests. The results showed a 20% reduction in whole-body insulin sensitivity and a 50% reduction in insulin clearance with all 3 methods; there was a negative association between insulin clearance and HbA1c supporting a role of chronic hyperglycemia. Subcutaneous administration of insulin, with the contribution of reduced clearance, abolishes the diurnal fluctuations of plasma insulin levels establishing an inflexible hyperinsulinemia (141), and reverses the distribution of insulin in the systemic circulation resulting in higher peripheral than portal levels increasing dramatically the risk for hypoglycemia and fat storage/weight gain, and inducing or aggravating insulin resistance in the liver and peripheral tissues (137) (Fig. 4). In T1D, the daily insulin dose normalized to body weight was positively associated with insulin resistance highlighting the role of chronic exogenous hyperinsulinemia (49, 53).

In support of these reports, infusions of insulin to healthy people for 2 to 4 days to produce even mild increases in peripheral insulin levels were shown to decrease insulin-mediated muscle glucose disposal and glycogen synthesis by 20% to 40% (142, 143). In a similar study in healthy adults, elevation of plasma insulin within the physiological postprandial range impaired endothelium-dependent vasodilation in brachial/common femoral arteries due to oxidative stress (144). Likewise, subjects with benign insulinoma chronically exposed to moderate or marked endogenous hyperinsulinemia were shown to develop generalized insulin resistance leading to an increase in EGP and muscle proteolysis, and a decrease in peripheral glucose disposal (145).

A study was designed to address this question, whether iatrogenic peripheral hyperinsulinemia or hyperglycemia is mainly responsible for reduced muscle insulin sensitivity in T1D (146) by examining people without diabetes (euglycemia, normoinsulinemia), with glucokinase maturity–onset diabetes of the young (hyperglycemia, normoinsulinemia), and with overt T1D (hyperglycemia, hyperinsulinemia). Using multiple regression analyses it was found that hyperinsulinemia was a more important predictor for the presence of insulin resistance than hyperglycemia in T1D.

The significance of insulin dose per day and hyperglycemia in coronary artery disease morbidity and mortality was investigated in nonobese T1D individuals followed for 18 years (147). Lower doses of insulin (50 units/day) predicted cardiovascular morbidity more closely than mortality; conversely, higher levels of HbA1c (8.9%) predicted cardiovascular mortality more closely than morbidity, even after adjustment for possible confounders (BMI, diabetes duration, smoking, lipids, hypertension, renal function). As discussed by the authors, although hyperinsulinemia may be a risk factor for atheromatosis, a reasonable daily amount of insulin used for treatment and properly adjusted to diurnal glucose variability can attenuate the severity and progression of vascular damage. Notably, in people with T1D and detectable C-peptide responses, insulin required for treatment is significantly less facilitating the improvement of glycemic control and insulin sensitivity (148).

In summary, these reports underscore the significant contribution of iatrogenic hyperinsulinemia to induce insulin resistance in T1D leading to increased cardiovascular risk. A primary treatment target is therefore the increase in insulin sensitivity to keep insulin requirements and inappropriate hyperinsulinemia as low as possible (149).

### Glucagon, Islet Amyloid Polypeptide

Glucagon is a potent hyperglycemic hormone and plays a central role in mediating increased EGP by stimulating glycogenolysis/gluconeogenesis during fasting and is physiologically suppressed postprandially by direct insulin and glucose effects on the α-cells in the islet environment (150). In healthy individuals, an independent and inverse association between fasting plasma concentrations of glucagon and insulin-stimulated glucose disposal has been shown which remained significant after correction for BMI, age, and glucose tolerant status (151).

In T1D, fasting and postprandial glucagon levels may be elevated due to intra-islet insulin deficiency and the inability of exogenous insulin to suppress glucagon secretion (Fig. 4) (152). In children/adolescents with T1D, postprandial plasma glucagon levels have been reported to increase progressively over time following diagnosis and correlate positively with postprandial hyperglycemia and the decline in β-cell function (153-155). In people with T1D and insulin resistance, fasting plasma glucagon levels were not suppressed by insulin during euglycemic-hyperinsulinemia suggesting a contribution to insulin resistance (38, 39, 120); dose–response effects of glucagon infusions on the stimulation of EGP (156) and glucose variability (157) have also been reported (Fig. 3). Children and adolescents with newly diagnosed T1D (average HbA1c 11.2%, BMI 16.5 kg/m^2^) and adults with long-standing T1D (average HbA1c 7.5%, BMI 24.1 kg/m^2^) showed increments in glucagon responses to hyperglycemia after a liquid mixed meal (158) or a 50-g oral glucose load (159) irrespective of ambient glycemia. In these studies, the paradoxical increase in glucagon secretion by hyperglycemia was not related to changes in GLP-1 or GIP. Interestingly, islets isolated from normoglycemic glutamic acid decarboxylase autoantibody (GADA)–positive donors showed preserved stimulation of insulin secretion, but already lost suppression of glucagon secretion by hyperglycemia, supporting a defect in glucose sensing in α-cells at the early stages of islet autoimmunity prior to β-cell destruction (160). The glucagonostatic potency of GLP-1 was investigated in C-peptide-negative adults with long-standing T1D (average HbA1c 7.7%, BMI 29 kg/m^2^) by using stepped hyperglycemic clamps (steady-state glycemia 90 to 270 mg/dL) along with increasing infusions of GLP-1 (161). The results showed that α-cells were insensitive to the glucagon-suppressive effects not only of hyperglycemia but also of GLP-1. Administration of somatostatin analogs along with insulin to people with T1D suppresses glucagon levels, improves postprandial glucose responses/diurnal glucose fluctuations, reduces insulin requirements and peripheral hyperinsulinemia, increases the effects of insulin on the stimulation of glucose disposal/suppression of EGP by more than 2-fold, reduces NEFA/glycerol levels, and increases nonoxidative glucose metabolism (162-165). These reports underline the importance of suppression of glucagon for improving glucose profiles in T1D. Of note, somatostatin analogs may induce hyperglycemia and increase the risk for prediabetes, excluding them as a therapeutic option for T1D (166).

IAPP is a protein co-secreted with insulin in β-cells. This peptide contributes to the inhibition of glucagon secretion, delays gastric emptying/glucose absorption rates, acts as a satiety agent to limit caloric intake, and is important for the control of carbohydrate homeostasis (167). Studies in nonobese healthy individuals or people with or without obesity or T2D using hyperglycemic clamps showed that endogenous IAPP does not affect insulin sensitivity (168). In T1D, IAPP secretion is severely impaired both under fasting and postprandial conditions following the impairment of insulin secretion (167). Its replacement along with insulin in adolescents/adults with T1D was associated with postprandial suppression of excessive glucagon excursions and improved glycemic control (169).

### Lipotoxicity

The mobilization of triglycerides from the adipose tissue in the form of NEFA is a primary pathophysiological mechanism in the development of low-grade inflammation, oxidative stress, insulin resistance, and cardiovascular complications in metabolic diseases (60, 99, 170) (Fig. 3). Excessive lipid availability, attributed to oversupply or impaired capacity for lipid oxidation, leads to triglyceride accumulation in muscle, liver, heart, and pancreatic β-cells influencing cellular glucose uptake/utilization and their metabolic function (10). In T1D, resistance of lipolysis to insulin and increased NEFA release and oxidation have been described in several studies with euglycemic–hyperinsulinemic clamps (22, 38, 41, 46, 51, 52, 171) (Table 1).

Ectopic lipid accumulation is a relatively early event in the pathophysiology of insulin resistance in muscle and liver while inflammatory cytokine spillover from the dysfunctional adipocytes and tissue infiltration occurs later and contributes to the progression of hyperglycemia in conjunction with the reduction in β-cell function (172). In nonobese inadequately controlled (average HbA1c 7.7-8.6%) insulin-resistant individuals with long-standing T1D, intramyocellular triglyceride content measured by ^1^H-MRS (38) or biopsies (173) was increased by over 2-fold vs healthy controls, was inversely associated with insulin-stimulated glucose disposal during euglycemic–hyperinsulinemia and was independent of overall adiposity; these defects were similar to those observed in overweight individuals with T2D and their glucose-tolerant normal-weight first-degree relatives. In contrast, 2 studies (43, 99) in nonobese adequately controlled (average HbA1c 6.5-6.8%) insulin-resistant individuals with long-standing or newly diagnosed T1D showed no elevation of intra-myocellular lipid content measured by ^1^H-MRS. As discussed by the authors, intramyocellular lipid content in T1D seems to depend on glycemic control rather than hyperinsulinemia or diabetes duration.

Hyperglycemia contributes to the accumulation of toxic lipid metabolites leading to protein kinase-C activation (PKC-ε isoform, with no changes observed for PKC-α, PKC-δ, and PKC-θ isoforms) and inhibition of insulin signaling, glucose transport, and insulin-mediated vasodilation, supporting the concept of glucolipotoxicity (174, 175). The intracellular accumulation of lipids may require the presence of hyperglycemia in the liver and β-cells, and the presence of hyperglycemia/hyperinsulinemia in muscle, leading to insulin resistance and β-cell dysfunction. Under these conditions, increased intracellular glucose metabolism increases the formation of malonyl-CoA a potent inhibitor of carnitine-palmitoyl transferase-1, the enzyme that mediates the transport of NEFA into the mitochondria to be oxidized, thereby favoring their storage in the cytosol and the formation of toxic products inhibiting glucose transport/metabolism (175-177).

**Figure 5.**
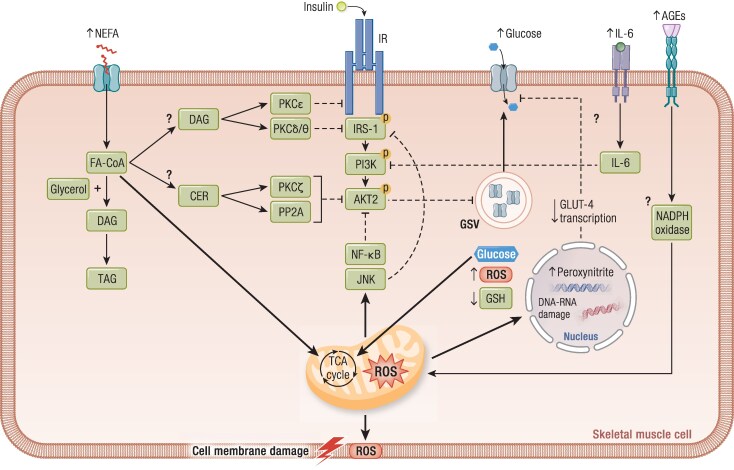
Proposed mechanisms of oxidative stress-mediated insulin resistance in muscle and adipose tissue in T1D. Glucotoxicity and lipotoxicity lead to the accumulation of AGEs and lipid metabolites inducing overproduction of reactive oxygen species (ROS) in mitochondria. Increased NEFA uptake leads to accumulation of toxic metabolites DAG and CER, which interfere with insulin signaling by nPKC and PCKζ/PP2A activation, respectively. On the other hand, increased NEFAs lead to TAG accumulation in skeletal muscle. Circulating cytokines, such as IL-6, inhibit PI3K in skeletal muscle directly inducing insulin resistance. Oxidative stress leads to cell membrane/protein damage and to NFkB/JNK pathway activation, which interferes with insulin signaling by inhibiting AKT2 decreasing insulin-stimulated glucose transport. Glutathione levels are decreased inducing insufficient antioxidant defense. This situation leads to increased peroxynitrite production and DNA/RNA damage resulting in decreased GLUT-4 translocation to the cell membrane aggravating insulin resistance (data from references 1, 206, 208, 211, 213). Abbreviations: IR, insulin resistance; IRS-1, insulin receptor substrate-1; PI3 K, phosphatidylinositol-3-kinase; DAG, diacylglycerol, TAG, triacylglycerol; CER, ceramide; IRS-1, insulin receptor substrate-1; NF-kB, nuclear factor-kappa B; JNK, c-Jun N-Terminal Kinases; AGE, advanced glycation end product; GSH, glutathione; NEFA, nonesterified fatty acid; nPKC, novel protein kinase C; FA-CoA, fatty acid coenzyme A; PP2A, protein-phosphatase 2; NADPH, nicotinamide adenine dinucleotide phosphate; GSV, GLUT4 storage vesicle. Mechanisms investigated in type 2 diabetes/insulin-resistant models but not in T1D are marked with question marks.

The sequence of events following NEFA oversupply to tissues has been comprehensively described by Roden and Shulman (1) using euglycemic–hyperinsulinemic clamps and [^31^P]- and [^1^H]-MRS. In the liver, activation of pyruvate carboxylate flux by NEFAs and the use of glycerol as a substrate stimulate gluconeogenesis and EGP; the development of insulin resistance further upregulates gluconeogenesis and reduces insulin-stimulated glycogen synthesis and glucose disposal aggravating postprandial hyperglycemia. In muscle, NEFAs and glycerol are rapidly esterified to triglycerides; accumulation of toxic NEFA metabolites (diacylglycerol, ceramides, fatty acyl CoA) interferes with insulin signaling at the level of insulin receptor substrate-1–associated PI3 kinase leading to a primary inhibition of insulin-stimulated GLUT-4 translocation and glucose disposal followed by a subsequent reduction of glycogen synthesis and glucose oxidation inducing insulin resistance (Fig. 5). Toxic lipid metabolites can also lead to stimulation of inflammatory pathways and oxidative stress (60, 62, 178-180).

Lipids are known to be involved in inflammation and β-cell destruction during T1D development (181). A lipidomic analysis in plasma of children and adolescents with new-onset T1D followed for 1 year showed that the levels of 9 diacylglycerol, 8 triacylglycerol, and 7 cholesteryl ester species after diagnosis could predict a decrease in meal-stimulated C-peptide and loss of β-cell function after 6 months (182). Sphingolipids (sphingomyelin, ceramides with their precursors and metabolites) were also associated with decreased C-peptide responses after 3, 6, and 12 months. Lipidomic analysis could also identify people with T1D and insulin resistance in nondiabetic overweight/obese individuals with insulin resistance (euglycemic–hyperinsulinemic clamps). Another lipidomic analysis in plasma and skeletal muscle revealed that insulin resistance was associated with higher diacylglycerol, triacylglycerol, ceramide, and lower lysophosphatidylcholine species (183). In muscle, insulin resistance was associated only with higher C18:0 sphingolipids.

Like hyperglycemia, elevated NEFA levels impair endothelial function and induce vascular insulin resistance in people with T1D by many of the mechanisms also involved in NEFA-mediated insulin resistance in muscle (184). NEFA metabolism by endothelial cells downregulates insulin signaling and impairs insulin-mediated vasodilation by decreasing the production of NO leading to endothelin-induced vasoconstriction and tissue hypoxia (185). In adolescents and young adults with T1D (average HbA1c 9%, diabetes duration 13 years) blood flow rates in the brachial artery were 20% lower, and plasma triglycerides and low-density lipoprotein (LDL)-cholesterol were both higher compared to healthy individuals. Decreased blood flow rates were inversely associated with LDL-cholesterol and diabetes duration but not with HbA1c levels (186). In well-controlled adults with T1D (average HbA1c 7%, diabetes duration 16 years) serum concentrations of adhesion molecules ICAM and VCAM were increased by 30% vs healthy controls. ICAM and VCAM levels were not correlated with HbA1c; VCAM concentrations were associated positively with LDL-cholesterol levels only in people with T1D and diabetic microangiopathy (187). In the vascular endothelial and smooth muscle cells, NEFA can increase the formation of ROS, induce oxidative stress and vascular inflammation (188), trigger the rate of apoptosis, and inhibit cell cycle progression (189, 190). NEFA-induced impairment of endothelium-dependent vasodilation in the forearm and oxidative stress can be corrected by intravenous administration of the antioxidant vitamin C (191).

In summary, these studies support the importance of lipotoxicity in the development of metabolic and vascular insulin resistance in T1D and the detrimental effects of NEFA in the impairment of endothelial function and progression of atherosclerosis and cardiovascular disease (CVD).

MASLD is defined as lipid accumulation in the liver in the absence of alcohol consumption (192). Specific lipid metabolites, such as ceramides, relate to whole-body but not hepatic insulin resistance as assessed by hyperinsulinemic–euglycemic clamps as well as to increased oxidative stress, and pro-inflammatory markers in obese individuals and play a central role in the development of metabolic dysfunction-associated steatohepatitis (193-195).

In T1D, ectopic lipid accumulation and MASLD development can result from obesity or altered insulin kinetics representing another independent risk factor for diabetes-related complications (196). The prevalence of MASLD was estimated to range from 12% to 47% varying broadly depending on study design, referral bias, and diagnostic modality. A recent meta-analysis reported a pooled MASLD prevalence of 19.3% (197, 198), which may associate with total daily insulin dose, VAT volume, and a positive family history of T2D (197, 199, 200). In contrast, MASLD prevalence was lower even in overweight individuals with long-standing inadequately controlled (average HbA1c 8%) T1D (8.8%) when compared with T2D (76% in insulin-naïve and 62% in insulin-treated) (201). The authors discuss the possibility of greater suppression of adipose tissue lipolysis by iatrogenic hyperinsulinemia leading to lower NEFA efflux and subsequently lower hepatic triglyceride synthesis in these persons with T1D. Also, nonobese insulin-resistant people with recent-onset, well-controlled T1D (average HbA1c 6.2-6.5%) of the GDS cohort showed a MASLD prevalence of 1.6% to 7% as measured by MRI and ^1^H-MRS methods, which was not different from healthy humans and 5-fold lower than in overweight/obese people with recent-onset T2D (98, 136).

In insulin-resistant individuals with inadequately controlled T1D (39) (Table 1) intrahepatic lipid content was 30% lower, whereas lipid oxidation and the ratio of glucagon/estimated portal insulin levels were 3-fold and 2-fold higher, respectively, vs healthy controls. Intrahepatic lipid content was not associated with hepatic or peripheral (muscle, adipose tissue, vascular endothelium) insulin resistance. These studies suggest that in T1D, in contrast to the definite involvement of lipid accumulation in muscle, there is no strong evidence for the association between hepatic insulin resistance and ectopic lipid accumulation within the liver. In the GDS cohort (98) hepatocellular lipid content, hepatic phosphorus metabolites (γATP, inorganic phosphate/Pi), and insulin sensitivity were estimated in adults with T1D at onset and after 5 years of follow-up. Despite increases in body weight, subcutaneous adipose tissue volume, and insulin resistance, T1D participants did not develop liver steatosis during follow-up although hepatic γATP and Pi decreased by 10% and 30%, respectively. Another study in the same cohort implied that lower hepatic ATP concentrations may be attributed to variations in genes involved in mitochondrial biogenesis, especially in peroxisome proliferator–activated receptor (*PPAR*) genes (202). These results imply that altered hepatic energy homeostasis develops early during the progress of T1D, occurs independently of hepatic lipid accumulation, and may be attributed to deterioration of glycemic control, iatrogenic hyperinsulinemia, and insulin resistance.

### Abnormalities of Mitochondrial Function and Oxidative Stress

Mitochondria play a significant role in fuel metabolism, cellular respiration, and insulin action/secretion in skeletal muscle, adipose tissue, liver, heart, vascular endothelium, and pancreatic β-cells being the site for glucose and NEFA oxidation through the tricarboxylic acid cycle (Fig. 3). Mitochondrial oxidative phosphorylation capacity, content per cell, and plasticity are important determinants of functional efficiency to ensure metabolic flexibility, the ability of muscle to rapidly switch between insulin-stimulated glucose and lipid oxidation to adapt metabolic demands to substrate availability and maintain energy homeostasis under conditions of fasting, feeding or exercising (203, 204). The higher capacity of muscle for metabolic switching depends on the metabolic characteristics of the individuals such as the levels of leanness/fatness, physical fitness, and insulin sensitivity (204, 205). In obesity and T2D, metabolic inflexibility has been suggested as a relevant determinant of insulin resistance and refers to a higher reliance of muscle upon glucose than fat oxidation during fasting and a higher reliance upon fat than glucose oxidation after meals (204). During euglycemic-hyperinsulinemia, glucose oxidation was shown to increase 7-fold in glucose-tolerant humans but only 4-fold in insulin-resistant individuals with T1D; basal lipid oxidation was 3-fold higher in the T1D participants and, in contrast to controls, it was marginally suppressed by insulin suggesting metabolic inflexibility (206).

Oxidative stress, a state resulting from the imbalance between the mechanisms of ROS production and antioxidant defensive molecules, is a crucial factor in impaired metabolic regulation. In situations of a decreased mitochondrial oxidative capacity and/or increased NEFA and glucose availability, oxidative stress is a determinant trigger for insulin resistance in muscle, adipose tissue, and liver; ROS-induced insulin resistance may be mediated by activation of the JNK and NF-κB pathways (207, 208) (Fig. 5). Oxidative stress has been proposed as a common cellular pathogenetic mechanism linking insulin resistance with impaired β-cell and endothelial function eventually leading to impaired metabolic regulation and CVD (common soil hypothesis) (131, 209). In muscle, glucotoxicity, lipotoxicity, and hyperinsulinemia promote mitochondrial ROS production, which can aggravate insulin resistance by further reducing insulin-mediated translocation of GLUT-4 transporters to the surface membrane thus contributing to the inhibition of glucose disposal (60, 210-212) (Fig. 5). Pancreatic β-cells do not depend on insulin for glucose transport and, therefore, glucose and NEFA overload increase proportionally their intracellular levels and ROS production leading to impaired insulin secretion (209).

In adults with T1D and optimal glycemic control for at least 1 year, insulin-stimulated glucose disposal and flux through ATP synthase were both reduced by 50% and 30%, respectively, vs healthy controls. Insulin-stimulated flux through ATP synthase was associated negatively with insulin resistance and HbA1c. The impairment of insulin action and mitochondrial ATP production was attributed to hyperglycemia/glucotoxicity, iatrogenic hyperinsulinemia, or previous inadequate metabolic control justified by the long duration of T1D (metabolic memory effect) (43). A further study (214) examined the association between insulin resistance and mitochondrial function in the muscle of insulin-resistant adolescents with T1D before and after moderate and submaximal leg exercise. Fasting serum NEFA levels failed to suppress during euglycemic-hyperinsulinemia. During exercise, ADP to ATP conversion and oxidative phosphorylation were lower by 25% and 57%, respectively, in people with T1D than in healthy controls, suggesting mitochondrial dysfunction; these defects were not associated with HbA1c, BMI, serum NEFA/triglycerides, inflammatory markers, and intramyocellular lipid content. However, there was a strong and independent positive association between insulin resistance and impaired mitochondrial function. These studies suggest that mitochondrial function in response to insulin or exercise is impaired in T1D and this defect may occur early in the progress of the disease. In conclusion, impaired mitochondrial function has been associated with insulin resistance. Acquired metabolic changes, such as hyperinsulinemia, hyperglycemia, and hyperlipidemia may promote ROS production leading to mitochondrial and cell damage in insulin-sensitive tissues and cells.

Following the results of large epidemiological trials in T1D/T2D and their follow-up, the term “metabolic memory” was given to the long-term benefits/harms of previous periods of optimal/suboptimal glucose regulation, suggesting that metabolic changes may be remembered in the target tissues for a long time after they occur (215). These reports provided evidence that tissue defects induced after short periods of impaired metabolic regulation can persist even if metabolic regulation follows. Mitochondria play a key role in metabolic memory through ROS overproduction and persistence of inflammatory responses and insulin resistance (216). In adipocytes incubated in vitro at high glucose concentrations or isolated from streptozotocin-treated hyperglycemic mice, ROS, IL-6 production, and PKC-δ activity were markedly increased, and insulin-stimulated glucose transport and phosphorylation were reduced indicating insulin resistance. These defects persisted for days after incubation of adipocytes at normal glucose concentrations or treatment with antioxidants suggesting post-translational modifications of signal transduction components (217). Transient periods of hyperglycemia can induce long-term epigenetic reprogramming of gene expression and activation of circulating monocytes and macrophages to enhance inflammatory cytokine release and establish an inflammatory milieu accelerating insulin resistance, atherosclerosis, and CVD (218). Transient intermittent hyperglycemia may also accelerate monocyte production in the bone marrow (219). The “metabolic memory” effect may explain, at least in part, the lack of an association between HbA1c and CVD risk observed in some of the studies in T1D (see “Clinical Relevance of Insulin Resistance”).

In T1D, episodes of excessive hyperglycemia and ketoacidosis are relevant metabolic complications, although their prevalence has decreased during the last few years due to new technologies, especially continuous glucose monitoring (220). In ketoacidosis, the stimulation of counterregulatory hormones generates insulin resistance in the liver and peripheral tissues leading to increases in lipolysis and plasma NEFA levels, low-grade inflammation, and oxidative stress (221, 222). It is therefore possible that, in T1D, transient episodes of ketoacidosis, oxidative stress, and insulin resistance may be remembered in the target tissues in the long term and contribute to impaired metabolic regulation even during periods of adequate glycemic control.

## The Interplay Between Insulin Resistance and the Autoimmune Process in the Development of T1D

### Evidence that Autoimmunity Leads to Inflammation and Insulin Resistance

Insulin resistance could be also mediated by proinflammatory cytokines secreted by cells involved in immunity such as macrophages, monocytes, and lymphocytes infiltrating islets during disease progression (223).

Sustained production of TNF-α, a potent cytokine, can induce insulin resistance and impair metabolic regulation by several mechanisms. In the liver, TNF-α increases glycogenolysis/gluconeogenesis and EGP. In muscle, this cytokine decreases glucose disposal and glucose oxidation, increases glycogen breakdown, anaerobic glycolysis, and lactate formation, and increases proteolysis and amino acid efflux. In the adipose tissue, TNF-α decreases glucose disposal and lipogenesis and increases lipolysis and NEFA release. In the vascular endothelium, TNF-α increases the production of ROS and decreases NO formation and blood flow. TNF-α can also increase fat deposition in muscle and liver and contribute to glucagon hypersecretion by the pancreatic α-cells (224). In children/adolescents and adults with T1D, gene expression and plasma levels of TNF-α have been found elevated and positively associated with HbA1c and plasma triglyceride levels vs healthy individuals (225, 226). Within the first year after T1D diagnosis inflammatory cytokines including members of TNF pathway (CD5, CCL23, CST5, IL-10RB, PD-L1, TNFRSF9) have been linked with reduced kidney function, possibly by facilitating inflammatory and fibrotic processes (227). IL-6, an inflammatory pleiotropic cytokine, also contributes to β-cell destruction in T1D. Chronic elevations of IL-6 can induce insulin resistance in the liver by inhibiting glycogen synthase and activating glycogen phosphorylase thus increasing glycogenolysis and EGP, and in the adipose tissue by increasing lipolysis and NEFA production (213). In skeletal muscle, IL-6 has been shown to induce insulin resistance (euglycemic–hyperinsulinemic clamps) possibly by an increase in intramuscular NEFA uptake, accumulation of NEFA-derived metabolites, and inhibition of glucose disposal (228). In children/adolescents and adults with T1D, plasma levels of IL-6 were found to be increased independently of BMI and HbA1c levels (229, 230).

### Evidence for Insulin Resistance Preceding Autoimmunity

An alternative hypothesis is that the development of insulin resistance may, at least in part, precede and contribute to the development of the autoimmune process in individuals genetically susceptible to T1D. The issue that autoimmunity may not be the primary mechanism in the development of T1D has been investigated in children with a positive first-degree family history of T1D before the initiation of autoimmunity from the age of 4 months to 4 years by measurements of preprandial and postprandial glucose concentrations and insulin autoantibody titers to address the question of whether autoimmunity is the main driver of T1D or follows the initiation of β-cell destruction and impaired glucose regulation by other factors. The intriguing results showed a modest but highly significant hyperglycemia at 4 months followed by a progressive decrease to euglycemia between 12 and 18 months of age, increasing thereafter to the previous levels of hyperglycemia from 18 months to 4 years, 2 months prior to autoantibody positivity (231). The authors concluded that β-cell dysfunction and impaired glucose regulation, at least at this age range, may precede the development of autoimmunity which seems to be a secondary event in the pathogenesis of T1D; the mechanisms related to autoimmunity may play a more significant role later in this process to aggravate metabolic decompensation.

T1D is a highly heterogeneous disease and endotypes (subtypes) have emerged to describe distinct pathophysiological mechanisms. According to current evidence, endotype 1 predominates in children diagnosed with T1D before the age of 7 years, shows less heterogeneity, and is associated with features of a more aggressive disease and multiple islet autoantibody positivity leading to the destruction of the majority of β-cells by the immune-mediated attack. By contrast, endotype 2 shows wider heterogeneity, is usually associated with single islet autoantibody positivity, increases progressively after 7 years of age, and predominates in people with T1D diagnosed after puberty and in adulthood; in this endotype, insulin resistance emerges as a pathogenetic mechanism contributing to β-cell destruction by the immune system (232, 233). A study in autoantibody-positive 1st-degree relatives with T1D showed that, at clinical diagnosis of T1D, participants with single autoantibody positivity were found to be older with higher insulin resistance (HOMA-IR) than those with multiple autoantibodies (234).

Even though the early rise in blood glucose levels suggests a primary β-cell defect (231), this association does not by itself establish a cause-effect relationship: a β-cell defect can lead to glucose intolerance but might also result from glucotoxicity effects of mild but persistent hyperglycemia on β-cells caused by other factors (89, 90). Although insulin resistance was not assessed in this study (231), a possible involvement—at least to some extent—cannot be excluded since BMI was positively associated with the level of hyperglycemia, and glucagon levels would be expected to be elevated in these participants due to β-cell deficiency (see “Glucagon, Islet Amyloid Polypeptide”). In islets isolated from normoglycemic glutamic acid decarboxylase/GADA-positive donors before clinical development of T1D, the suppressive effect of hyperglycemia on α-cells was lost before β-cell failure supporting an early defect (160, 235). Furthermore, even in healthy glucose-tolerant individuals, sustained mild physiologic hyperglycemia has been shown to impair β-cell function and induce insulin resistance (236). Hyperglycemia can also stimulate the production of autoantigens such as GADA a primary β-cell antigen involved in the autoimmune process leading to β-cell destruction (237).

The factors leading to increased vulnerability and damage of β-cells in the physical history of T1D before seroconversion may be related to genetic predisposition, metabolic stress, impaired islet function due to reduced pancreas volume, viral infections (such as coxsackie), and environmental factors (238, 239). Of these factors, endocrine-disrupting chemicals, such as dioxins, bisphenols, and phthalates can accumulate in the trophic chain throughout generations, are stored in adipose tissue, and are released into the circulation during lipid mobilization over the lifetime. Exposure of the fetus during pregnancy and the newborn after birth and onwards can directly induce islet inflammation, β-cell destruction, and immune system mobilization, and hence promote the development of T1D in genetically predisposed individuals (240, 241). Various studies confirmed the deleterious role of these chemicals but also the chronic exposure to air pollutants for development of insulin resistance via pulmonary and systemic inflammation, oxidative stress, and endoplasmic reticulum stress (242, 243). However, their role in the development of insulin resistance in T1D still needs to be examined. Also, viral infections are associated with the development of severe and long-lasting resistance of muscle glucose disposal and glycogen synthesis to insulin (euglycemic–hyperinsulinemic clamps, glucose tracer infusions, indirect calorimetry) (95).

Under physiological conditions, native insulin and its precursor molecules can act as primary autoantigens due to translational/post-translational modifications occurring during the secretory process (244), and fragments of pre-proinsulin in people with T1D can be targets of the CD8+ cytotoxic T-cells (245). In the presence of insulin resistance, the demand for a compensatory increase in insulin secretion from β-cells with a genetically determined high rate of apoptosis early in the physical history of T1D, in addition to cellular stress, would further increase the expression of these insulin/proinsulin molecules and antigen presentation to the immune system, and hence may trigger autoimmunity, inflammation, and β-cell destruction and death (246, 247).

In summary, the pathophysiology of T1D is likely multifactorial; while almost exclusively driven by autoimmunity in some cases, other factors may play a more important role in in the destruction of β-cells and initiation of autoimmunity in other cases. Based on current experimental evidence, insulin resistance may indeed contribute to the development and progression of T1D in the context of genetic predisposition to autoimmunity operating as an assisting factor. Likewise, insulin resistance could play a more active role in the initiation of autoimmunity but this possibility needs to be further investigated. Nevertheless, causality in the interplay between autoimmunity and insulin resistance is still uncertain (5, 238, 248).

## Clinical Relevance of Insulin Resistance

The risk of CVD and related mortality in T1D has been reported as lower (249) or higher (250) than in T2D, and its development and progression are tightly connected to insulin resistance (8, 103). Hyperinsulinemia, dyslipidemia, hypertension, weight gain, smoking, and sedentary life are all major risk factors linked by metabolic and vascular insulin resistance as a common pathogenetic mechanism to induce endothelial dysfunction and vascular damage leading to vascular complications regardless of the presence of diabetes (251, 252). Notably, hyperglycemia can also lead directly to vascular pathology and complications through oxidative stress-related mechanisms (8, 104, 131) (see “Hyperglycemia, Glucotoxicity, and Glucose Variability”) (Fig. 3). In 2 epidemiological T1D studies, all-cause mortality (253) or coronary artery disease (254) showed positive associations with other factors related to insulin resistance, such as increased proinflammatory markers, lipid levels, blood pressure, kidney disease, and low physical activity level, but not with HbA1c. The authors suggested that these discrepancies could be explained either by the small number of cardiovascular events (253) or by the hypothesis that even though hyperglycemia may be associated with more extensive atherosclerosis, such as in lower extremity arterial disease, it may show only modest associations with coronary heart disease (254, 255). In 1 of these studies (254) eGDR was inversely correlated with coronary artery disease suggesting insulin resistance. In adults with T1D, even at levels of HbA1c 6.9% or lower suggesting adequate glycemic control, cardiovascular deaths were 2-fold higher than in age/BMI-matched healthy glucose-tolerant people (256). Diabetes-associated complications, especially nephropathy and retinopathy are frequent already within the first year after T1D diagnosis (257).

Insulin resistance can promote atherogenesis via multiple mechanisms. In the adipose tissue, insulin resistance increases lipolysis and NEFA influx to the liver, muscle, and vascular endothelium (lipotoxicity) and stimulates the release of pro-inflammatory cytokines leading to impairment of insulin signaling in these tissues; these effects aggravate oxidative stress and hyperglycemia (see “Contribution of Individual Tissues to Insulin Resistance”) (Fig. 3). Obesity is a significant driving force for adipose tissue insulin resistance (see “Obesity”). Insulin resistance and hyperinsulinemia owing to exogenous insulin contribute to the elevation of blood pressure—a significant risk factor for endothelial damage—via inhibition of the NO pathway in the endothelium, and stimulation of sympathetic nervous system activity, smooth muscle growth, and sodium retention (258). Of note, elevated blood pressure is more prevalent in people with T1D than in healthy humans (259). Furthermore, hyperinsulinemia alone can induce insulin resistance (see “Hyperinsulinemia”).

Several studies investigated the association of insulin resistance assessed by euglycemic–hyperinsulinemic clamps, with diabetes-related complications in T1D most of them suggesting independent effects.

In individuals with T1D followed for 3 years, insulin resistance preceded and was positively associated with the development of microalbuminuria; this association was independent of diabetes duration, BMI, insulin dose, systolic/diastolic blood pressure, and HbA1c. Notably, 86% of the T1D individuals with microalbuminuria had first- or second-degree relatives with T2D (34). In a similar study in T1D (29), insulin resistance was also positively correlated with microalbuminuria. Diabetes duration, HbA1c, and microalbuminuria, but not BMI, body fat percentage, and age were inversely associated with forearm glucose disposal supporting a pathogenetic role of chronic hyperglycemia. Studies that measured eGDR and insulin sensitivity scores in persons with T1D also found reverse associations with retinopathy and its severity, and/or kidney disease (260, 261).

A study in adolescents/young adults with T1D (50) investigated the role of insulin resistance as an early factor for the development of carotid-intima media thickness (IMT). There was an inverse association between glucose disposal and carotid-IMT regardless of BMI and waist circumference, HbA1c, blood pressure, total and high-density lipoprotein (HDL)/LDL-cholesterol, triglycerides, and high-sensitive C-reactive protein in both T1D participants and healthy controls. However, this association was left-shifted in T1D indicating that at any given value of carotid-IMT, T1D individuals were more insulin-resistant than controls. In agreement with other authors (37, 49, 52), these results suggest that insulin resistance is the most significant and independent factor associated with the development of atherosclerosis in T1D and cannot be predicted by other classic risk factors. However, regarding muscle insulin resistance, a heterogeneity between muscle types and their association with coronary artery disease has been described in T1D (36, 43, 53), indicating that this association seems to be far more complex than previously thought, and may be related, at least in part, to the intrinsic metabolic characteristics of the individual fiber types and especially their glycolytic rather than oxidative properties (53).

Insulin action, cardiopulmonary fitness during exercise, and cardiovascular function were examined in lean, relatively well-controlled adolescents with T1D (41). Insulin-mediated glucose disposal and blood flow in muscle were lower, total cholesterol, LDL, and adiponectin levels were higher, and BMI, fat distribution, and inflammatory markers were not different from healthy controls. Typical risk factors for insulin resistance and CVD were absent, and cardiac indexes revealed diastolic dysfunction/left ventricular hypertrophy despite short diabetes duration. Insulin-stimulated glucose disposal and forearm blood flow but not HbA1c or diabetes duration were positively associated with peak oxygen consumption during exercise indicating that insulin resistance has effects independent of hyperglycemia to reduce exercise capacity in these individuals. The authors (41) concluded that insulin resistance is strongly and independently associated with impaired exercise capacity and cardiovascular dysfunction in T1D and can be present from the early stages of disease progression.

In people with T1D without vascular complications, the responsiveness of microvasculature to vasoconstrictive stimuli is exaggerated owing to an activation of the sympathetic nervous system, and arterial stiffness is not decreased during euglycemic-hyperinsulinemia as observed in healthy individuals (32, 33, 54, 262). Whole-body glucose disposal was inversely associated with arterial stiffness in both T1D and healthy controls supporting a causal effect of the degree of insulin sensitivity. In the individuals with T1D, HbA1c was positively correlated with arterial stiffness suggesting a role of chronic hyperglycemia. These defects, along with inappropriate hyperinsulinemia, may predispose insulin-resistant people with T1D to the development of hypertension (32).

Overall, these observations suggest that, despite a profound insulin resistance estimated by euglycemic–hyperinsulinemic clamps and its association with adverse clinical outcomes, the criteria normally used for prediction in the general population or people with T2D, may not always apply to T1D. These individuals seem to have a clinical/biochemical phenotype less consistent with the metabolic syndrome; its components may not accurately predict CVD and may also underestimate the important, and potentially independent contribution of insulin resistance to its pathogenesis (263). Insulin resistance has been suggested to provide the missing link between T1D and CVD (41) and, therefore, surrogate markers, such as the eGDR, and their association with diabetes-related complications should be interpreted with caution.

These conclusions are further supported by studies examining the association of insulin resistance-related risk factors with cardiovascular outcomes and mortality. In 33.333 adults with T1D and 166.529 healthy individuals from the general population, all major cardiovascular outcomes (mainly myocardial infarction and heart failure hospitalizations) were higher in T1D than controls, even when HbA1c, blood pressure, smoking, albuminuria, and LDL-cholesterol were improved suggesting an independent involvement of insulin resistance (264). A further study (265) investigated the association between subclinical atherosclerosis and obesity, hyperglycemia, hyperinsulinemia, low HDL-cholesterol, hypertriglyceridemia, and hypertension evaluated alone and in various combinations. The results showed independent positive associations between these combinations and carotid intima-media thickness indicating a synergy for atherosclerosis risk beyond what would be expected from individual effects. As the authors concluded, therapeutic interventions targeting insulin resistance as a common soil mechanism may have benefits on CVD prevention beyond those expected from individual treatments of risk factors. Furthermore, in individuals with T1D, markers of insulin resistance (hypertriglyceridemia, hyperglycemia, obesity, smoking, diabetes duration) were shown to be strong and independent risk factors for the incidence of neuropathy and retinopathy; neuropathy incidence was also doubled by the presence of CVD at baseline (266, 267). In line with this, maintenance of near-normoglycemia over 24 years after diagnosis was associated with complete prevention of confirmed polyneuropathy in lean people with T1D (268). In nonobese inadequately controlled (average HbA1c 8%) adults with T1D, arterial wall inflammation, monocyte/macrophage activity in bone marrow and spleen (both assessed by 2-deoxy-2-[^18^F]-fluoro-D-glucose positron emission tomography) and proinflammatory circulating biomarkers were higher, independent of HbA1c, compared with healthy controls (269).

Residual insulin secretion in overt T1D can be maintained for decades after diagnosis depending on metabolic control and may be important for favorable clinical outcomes (270). Preserved β-cell function contributes to the improvement of insulin sensitivity, facilitates optimal glycemic control, and insulin dose reduction, and reduces the risk for hypoglycemia, ketoacidosis, retinopathy, nephropathy, hypertension, dyslipidemia, and CVD (148, 271-273).

The heterogeneity and complexity of T1D diabetes have underlined the need to identify disease subtypes (234). In the GDS (274) individuals with recent onset T1D/T2D were characterized according to the new diabetes classification clusters as previously proposed (275). The cluster with severe autoimmune diabetes (SAID) defined by poor metabolic control, relatively low BMI, insulin deficiency, and positive autoantibodies was differentiated from the other 4 subtypes based on phenotypic and pathophysiological characteristics. During the 5-year diabetes progression, 82% of the individuals in SAID remained in the same cluster whereas 18% moved to severe insulin-deficient diabetes (SIDD), mild obesity-related diabetes (with low insulin resistance, MOD), and mild age-related diabetes (MARD, individuals older than in other clusters but similar to mild obesity-related diabetes with mild metabolic derangements) clusters sharing their characteristics. Interestingly, the SAID cluster had the highest risk for early diabetic neuropathy and retinopathy whereas the SIRD cluster (severe insulin-resistant diabetes) showed an increased risk for nephropathy (274, 275). The authors discuss that insulin resistance leads to increased salt sensitivity, glomerular hypertension, and hyperfiltration independently of glycemic control. On the other hand, insulin seems to have neuroprotective effects and specifically in the retina reduces retinal cell apoptosis, glial activation, and VEGF upregulation (276), and its deficiency is associated with retinopathy progression (277). This classification and the ongoing investigation of T1D progression between clusters will allow more precise and targeted therapeutic interventions in people with T1D and thereby lead to improved strategies for the prevention of chronic diabetes-related complications (278).

## Possible Risk Factors for Insulin Resistance

### Obesity

Although obesity used to be a rare phenomenon in T1D, in recent years it has represented a rising problem, especially in younger ages, with increasing clinical significance due to its association with insulin resistance (279, 280). In the T1D Exchange Registry US Study (26.697 participants, age range 1-93 years), the prevalence of overweight or obesity was 49% (281). In children and adolescents with T1D, the prevalence of overweight/obesity in boys and girls has been reported at 22.3% and 27.2%, respectively, and was associated with an atherogenic lipid profile (282, 283). Two of the predictive factors for these changes were inadequate glycemic control and intensified insulin therapy with multiple injections (279). The association between BMI and incident T1D in 1.5 million healthy children/adolescents followed until diabetes onset showed that overweight/obesity was highly associated with an increased risk for T1D development in early adulthood (284). Α Mendelian randomization study revealed that greater childhood adiposity associates directly with an increased risk of T1D (285). Of note, a reduction of the proportion of children within the highest obesity category by 10% could result in a 22% reduction in T1D cases.

Several factors may differentiate lipid accumulation and weight gain between individuals with T1D and their peers with normal glucose metabolism.

Important examples of genes related to obesity that are also involved in T1D and associated with greater weight gain and faster disease progression are the fat mass and obesity-associated (FTO) gene and gene-encoding the melanocortin-4 receptor (MC4R); the function of both genes is related to appetite regulation and energy expenditure (286, 287). Data in persons with T1D showed an association between worse eGDR with the presence of A allele of rs12970134 near the MC4R gene but not with polymorphisms in FTO gene (288). Epigenetic modifications, such as methylation and acetylation seem also to play an important role by modifying genetic pathways in response to environmental exposure to obesity in T1D (289) (eg, exposure to maternal obesity and diabetes or early postnatal overnutrition) (290). In this study, in utero exposure to maternal hyperinsulinemia was associated with altered hypothalamic gene expression and progression to insulin and leptin resistance favoring juvenile obesity. In this context, the coexistence of T1D with obesity and other risk factors for CVD found in people with T2D should be discussed. These individuals have a family history of T2D, may be negative for T1D-related autoantibodies, have marked insulin resistance and require higher insulin doses for treatment, experience higher body weight for their age with central fat distribution, and show a worse lipid profile, and higher incidence of complications on intensified insulin therapy compared to people without such a family history. These findings underline a possible role of genetic background, that leads to genetic susceptibility to obesity, insulin resistance, β-cell dysfunction, and CVD (291, 292).

Peripheral insulin administration, mostly using multiple injections, and hyperinsulinemia are major reasons for conservation of calories, lipid accumulation, and weight gain. Additional underlying causes for the increasing incidence of overweight or obesity in T1D are frequent eating due to repeated episodes of hypoglycemia or preventive eating due to fear of hypoglycemia, and socioeconomic and cultural drivers (279, 293, 294). Further, important associated parameters are emotional aspects such as low self-esteem, chronic stress, frustrations, worries, and depression; these are all considered adaptive responses to disease burden, seem to play an important role in the development of unhealthy eating patterns, sedentary life, and social isolation, and can be associated with the development of insulin resistance (295). These factors work additionally to the obesogenic drivers within the physical activity and food landscape characterized by a tendency towards a sedentary life and increased availability/overconsumption of energy-dense foods/beverages, impaired sleep duration/quality, and urbanization of people within countries to which both the general population and individuals with T1D are exposed (293, 294).

Therefore, T1D-related drivers seem to contribute to weight gain, which in turn may accelerate β-cell autoimmunity and destruction creating a vicious cycle. Overweight and obesity are both associated with adipose tissue dysfunction, leading to overproduction of NEFAs and glycerol and in the long run a wide variety of proinflammatory cytokines which induce insulin resistance (see “Mechanisms”). In individuals genetically susceptible to T1D, this would be expected to increase the burden for insulin requirements inducing endoplasmic reticulum stress on β-cells and accelerate their apoptosis rendering them more immunogenic (296-298). In support of this suggestion, in single autoantibody-positive and low-genetic risk children and adolescents without a family history of T1D, high BMI was associated with progression to multiple autoantibodies accelerating clinical development of T1D (299). At this age, the development of T1D is particularly common due to puberty and insulin resistance (92-94). In children/adolescents with T1D insulin-stimulated whole-body glucose disposal (euglycemic–hyperinsulinemic clamp) was shown to decrease with the onset of puberty and in the post-pubertal state, and inversely correlate with adiposity markers, HbA1c, blood pressure, triglycerides, and insulin dose (300). In another study, following clinical diagnosis of T1D, increased body weight was associated with rapid disease progression to insulin deficiency in the age group 10-18 years. The authors discussed the possibility that insulin resistance could be involved in the pathophysiology of β-cell exhaustion due to pubertal transition (301).

In summary, in T1D, overweight/obesity may induce insulin resistance, and accelerate autoimmunity and β-cell destruction in individuals genetically susceptible to T1D. These results support the suggestion that prevention of weight gain could at least delay the occurrence of T1D.

### Sleep Disturbances

Sleep is crucial for inulin action and mammalian functions such as regulation of food intake and energy homeostasis. Altered sleep patterns (less total sleep time, frequent wake after sleep onset/fragmentation of sleep, and late bed/early rise times) have an important impact on impaired metabolic regulation, insulin resistance, and cardiovascular risk, especially when combined with physical inactivity (302, 303). A study on healthy adults examining the relationship between sleep, obesity, and insulin action showed that overfeeding/weight gain–mediated insulin resistance in the liver, adipose tissue, and muscle (euglycemic–hyperinsulinemic clamps) was manifested only in participants with short sleep duration and not in those with longer sleep duration emphasizing the importance of sleep in insulin action and metabolic regulation (304).

In healthy people, even 1 or 6 nights of partial sleep deprivation vs normal 8-hour sleep induced marked resistance of EGP, whole-body glucose disposal, and lipolysis to insulin, stimulated counterregulatory hormone secretion (cortisol, catecholamines, thyroid hormones), increased NEFA production and their muscle oxidation rates (305-307), and impaired Akt phosphorylation by insulin in adipocytes (308). These results indicate that physiological sleep patterns are important for the functional integrity of tissues involved in metabolic regulation and their insulin responses.

Short sleep duration and disturbed sleep architecture are high among people with T1D with an estimated prevalence of up to 15% and have been related to higher HbA1c and nondipping patterns presenting a risk factor for retinopathy and nephropathy (309, 310). In people with T1D, even a single night of partial sleep restriction reduced the sensitivity of whole-body glucose disposal but not EGP to insulin by 18% during euglycemic–hyperinsulinemia (311). Poorer sleep in T1D has also been strongly associated with higher within-person and between-person glucose variability (312). Short sleep duration has been associated with increases in ghrelin, an appetite-stimulating hormone. On the other hand, morning levels of leptin were reduced after sleep deprivation and remained suppressed after sleep recovery. These hormonal changes may contribute to impaired insulin action (313).

Although obstructive sleep apnea is a significant problem in T2D, it has also been described in people with T1D especially males, older, and obese. Oxygen desaturation caused by sleep apnea, a situation seen together with snoring, leads to increased cortisol/catecholamine release, which in turn may contribute to insulin resistance and impaired glucose metabolism (314).

In summary, these data suggest that, in T1D, sleep disturbances can lead to significant insulin resistance. These individuals should be educated to optimize their sleep habits focusing on sufficient sleep duration and regularity since these could have beneficial effects on lifestyle, glycemic/lipid control, insulin sensitivity, and ultimately cardiovascular risk.

### Physical Inactivity

Physical activity/exercise is a powerful modulator of metabolic fluxes, stimulates glucose disposal independently of insulin, provides a safe and effective therapeutic regimen that protects from obesity and CVD, represents a cornerstone in a healthy lifestyle and, therefore, should start as early as possible in people with diabetes and continue across the lifespan (315).

In healthy individuals, physical inactivity (bed rest) for only 3 to 5 days was associated with hyperglycemia/hyperinsulinemia, insulin resistance (50% increase in HOMA-IR and 15% decrease in the insulin sensitivity index after OGTT), blood flow decreases in forearm muscles (venous occlusion plethysmography and ultrasound), and increases in blood triglycerides and systolic blood pressure, while blood levels of IL-6, TNF-α, and adiponectin remained unchanged (316). In studies using euglycemic–hyperinsulinemic clamps, a sedentary lifestyle was associated with insulin resistance in muscle (317) and adipose tissue (318).

Studies in children/adolescents with T1D have shown that these individuals are more sedentary, less active, and have lower cardiorespiratory fitness than their healthy peers; a sedentary lifestyle is also associated with inadequate glycemic control (319, 320). It has been suggested that a lack of physical activity could be responsible for triggering autoimmunity or accelerating the progression to overt T1D, but there is currently no supporting clinical data (321). In children aged 5-15 years positive for multiple autoantibodies, a study recently showed that every 10-minute increase in moderate to vigorous exercise per day could lower the risk of progression to overt T1D by 8%; as the authors concluded, the protecting effect of exercise might be attributed to the lowering of BMI and insulin resistance (322).

## Therapeutic Interventions Specifically Addressing Insulin Resistance

Due to the nature of T1D, glucometabolic control is more complex than T2D. Although novel basal/prandial insulin analogs are now available, over 70% of people with T1D remain at suboptimal glycemic control. On the other hand, intensified treatment increases the risk of hypoglycemia and body weight gain. Furthermore, in T1D, the intra-islet regulation of glucagon secretion by insulin is lost, and the prevalence of obesity, dyslipidemia, depression, and other risk factors for CVD is increased. The treatment of T1D is therefore staggering, and the way to tackle this is by assigning therapeutic targets not only within but also beyond glycemic control. This part of the review will focus only on strategies with regard to improving insulin resistance.

### Lifestyle Modifications

In T1D cohorts, exercise, and diet interventions have been judged to help improve insulin resistance, reduce daily insulin dose, and optimize glycemic control (323). The rates of glucose disposal in the contracting muscle during exercise increase independently of insulin owing to the increase in intrinsic activity and translocation of GLUT4 transporters from intracellular pools to the myocellular surface; the increase in glucose disposal with exercise against resistance is facilitated by increases in muscle mass and vascularization/blood flow (324-326).

In T1D, insulin resistance is associated with reduced exercise capacity (41). In insulin-resistant adults with T1D on CSII or multiple insulin injections, endurance exercise training (cycle ergometer, 1 hour per day, 4 times per week, or walking/jogging, ball games, gymnastics for 1 hour/day, 2-3 times per week) improved the sensitivity of glucose disposal to insulin (20-60%, euglycemic–hyperinsulinemic clamps) and aerobic capacity (8%), lowered total daily insulin dose (8-16%), increased HDL-cholesterol (6%), and increased the activities of mitochondrial enzymes (citrate synthase, succinate dehydrogenase) in muscle (20%), despite unchanged HbA1c levels (327, 328). In adolescents with T1D, insulin-stimulated glucose disposal (euglycemic–hyperinsulinemic clamps) and aerobic capacity were increased by 23% and 8%, respectively, while daily insulin dose and HbA1c were not reduced after endurance training (45-minute cycle ergometer sessions, 3 times per week, for 12 weeks) (329). In adults with T1D, individuals with higher residual C-peptide were reported to spend more time in euglycemia (continuous glucose monitoring) after a bout of moderate-intensity endurance exercise (330). As discussed, this could be explained by the combination of residual insulin secretion and the postexercise increase in insulin sensitivity. Resistance exercise in adults with T1D (heavy weight training, 3 days/week, 10 weeks) increased muscular strength, improved HbA1c and diurnal self-monitored glucose levels (16% and 10%, respectively), and lowered plasma triglycerides (10%) and daily insulin dosage (10%) (331).

Effects of endurance (45 minutes on a treadmill) and resistance exercise (45 minutes on several sets with weights) were compared in individuals with T1D (332). Blood glucose levels (160 mg/dL at baseline in both interventions) declined much less during resistance exercise (21%) than during endurance exercise (37%). However, in the postexercise period, glucose levels remained stable for hours at 120 mg/dL after resistance exercise, whereas they increased after aerobic exercise to the pre-exercise levels; these results may explain, at least in part, the lack of beneficial effects on HbA1c with aerobic training.

The improvement in insulin sensitivity by endurance training in T1D can increase the risk of hypoglycemia for many hours after the sessions, especially overnight. Explosive and resistance training increases the risk of hyperglycemia, whereas endurance/aerobic exercise can induce hypoglycemia (333-337). For these reasons, mixed exercise activities have been recommended in T1D; in this case, resistance should precede endurance sessions to ensure glycemic stability and avoid postexercise excessive hyperglycemia or hypoglycemia (323, 324). Resistance activities stimulate growth hormone secretion which, through the increase in EGP and lipolysis, protects from hypoglycemia during the following endurance sessions (338). Specifically for individuals on ultra-long-acting insulin pre-exercise fructose intake can effectively prevent training-induced hypoglycemia (339). In individuals with T1D participating in a high-intensity interval training (HIIT) program (324) catecholamine and growth hormone increase along with a decrease in skeletal muscle utilization suggesting a shift towards alternative fuel oxidation and reduced hypoglycemia risk with this training modality (340). In a meta-analysis, the long-term exercise and particularly the combination of endurance and resistance training in people with T1D showed improvements in HbA1c and lipid profile, and a reduction in insulin dose up to 0.88 units/kg/day (341).

Regarding exercise timing following meals in T1D, bouts of exercise (walking, moderate- or high-intensity exercise) in the morning, midday, or late afternoon/evening performed immediately, 2 hours or 2 to 24 hours postmeal can lead to blood glucose reductions depending on exercise intensity/duration and insulin requirements for treatment (342). Morning exercising may be particularly beneficial in T1D to improve early morning elevations in glucose due to insulin resistance (343).

In individuals with T1D, various combinations of endurance and resistance exercise 3 times per week for 8 weeks induced β-cell protection against endoplasmic reticulum stress, the main mechanism responsible for β-cell apoptosis during the progression of T1D; interestingly, this effect persisted for 2 months after the end of the training program (344). As discussed by the authors, if initiated early in life, exercise could be a useful nonpharmacological intervention to benefit antibody-positive individuals by delaying the onset of the disease.

Although physical activity benefits metabolic control and cardiovascular risk factors in T1D (345), a major barrier to regular participation is fear of exercise-induced hypoglycemia (346). In adults with T1D on CSII or multiple daily injections, postprandial glucose responses, time above range, and glucose variability (continuous glucose monitoring) were reduced by about 16%, 15%, and 7%, respectively, while time in range was increased by about 19% without hypoglycemia when participants performed 3-minute bouts of light-intensity walking at 30-minute intervals starting 60 minutes after meal initiation, for 7 hours. Glycemic improvements were sustained for 2 days after the end of the study under free-living conditions (347). Thus, moderate physical activity in individuals with T1D may be useful not only to improve glycemia but also to increase insulin sensitivity without risking hypoglycemia. Furthermore, these simple interventions may encourage inactive people with T1D who are unwilling to be involved in structured programs to incorporate physical activity into their everyday schedule (348).

### Modes of Insulin Treatment

CSII is a well-established tool to improve glycemic control of poorly regulated people with T1D on multiple daily injections of insulin by establishing a more optimal way of insulin delivery. CSII treatment of insulin-resistant individuals with T1D increased insulin-stimulated glucose disposal by 30% during euglycemic-hyperinsulinemia, and reduced HbA1c, self-monitored blood glucose levels, and daily insulin requirements by 23%, 30%, and 20%, respectively, vs multiple daily insulin injections (MDIs). However, to the best of our knowledge, cross-over trials using euglycemic–hyperinsulinemic clamps allowing to compare the 2 regimens are lacking. Possible mechanisms for improved insulin sensitivity were postulated to be improved glycemic control and lower total insulin dose, resulting in reduced systemic hyperinsulinemia (13-15, 18, 19, 349). In 18.168 people with T1D from the Swedish National Diabetes Register followed for 6.8 years, a study investigated the association between CSII treatment and cardiovascular mortality and showed that CSII reduced fatal coronary heart disease, fatal CVD, and all-cause mortality by 45%, 42%, and 27%, respectively vs intensified insulin treatment with MDIs (350). As discussed by the authors, the results may be explained by more stable glycemia and lower glucose fluctuations, lower hypoglycemia risk and duration/frequency of hyperglycemia, and less amount of insulin units for treatment. Although these parameters can mediate insulin resistance, this study did not propose a direct link between CVD and improved insulin action. Likewise, the transfer of people with T1D to CSII from multiple insulin injection regimens was accompanied by a reduction in systolic and diastolic blood pressure by 4 and 2 mmHg, respectively (351).

In a joint study from Steno and Joslin Diabetes Centers on 1258 adults with poorly controlled T1D 1 year after switching from MDIs to CSII, HbA1c was significantly improved with no weight gain; the main predictor of HbA1c decrease was higher HbA1c at baseline (352). Weight gain during CSII may be associated with the percentage of basal rather than prandial insulin: the reduction of basal insulin to less than 40% of the total with appropriate increases in prandial insulin was shown to induce less weight gain independently of activity levels (353). A study of 3.887 Swedish children followed for 24 months after onset of T1D showed that CSII vs MDI and residual insulin secretion were associated with a higher frequency of partial remission of diabetes (354). Notably, T1D remission has been associated with higher insulin sensitivity measured with euglycemic–hyperinsulinemic clamps (55, 56).

### Metformin

Several therapeutic agents used in T2D to improve insulin sensitivity have been tried in T1D (355). Metformin, the traditional first-line drug for T2D, shows significant glucose-lowering and insulin-sensitizing properties by several still incompletely understood mechanisms, including reduction of hepatic gluconeogenesis, stimulation of muscle glucose disposal in muscle, improvement of lipid metabolism by promoting NEFA re-esterification and inhibiting lipolysis and modulating gastrointestinal function (356). Following a 6-month period of adding metformin 850 mg daily to CSII therapy in T1D, a reduction in insulin requirements and total/LDL-cholesterol was observed despite unchanged glycemic control (357). A systematic review including 9 intervention studies with metformin in T1D concluded that metformin addition reduced insulin requirements and HbA1c by 0.6% to 0.9%, and facilitated weight loss while being well-tolerated (358). However, long-term effects after 10 years of follow-up failed to show sustained improvements in HbA1c, insulin dose, or weight loss (359). This was also a finding of the REMOVAL trial, which examined the cardiovascular effects of metformin after 3 years of therapy in T1D. Although improved HbA1c and total insulin requirements were not sustained after 3 months of therapy, carotid-IMT showed a significant reduction indicating possible cardiovascular benefits (360). Metformin (1000 mg twice a day, 3 months) added to insulin in insulin-resistant overweight or lean adolescents/young adults with T1D improved whole-body insulin resistance, reduced body weight/fat mass and daily insulin dose, and improved vascular health, without affecting HbA1c (361-363). Episodes of lactic acidosis with metformin were not reported in any of these studies. Altogether these reports, although promising, do not support a general use of metformin in T1D (364).

### Thiazolidinediones

Pioglitazone, a potent insulin sensitizer, enhances insulin action in muscle by activating PPARγ receptors and is widely used in T2D treatment to reduce insulin resistance (355). Administration of pioglitazone (30 mg once daily) together with insulin (49 units/day) to people with T1D (HbA1c 7.2%, fasting and postprandial plasma glucose 117 mg/dL and 164 mg/dL, respectively, BMI 19.6 kg/m^2^, diabetes duration 7 years) for 24 weeks lowered HbA1c and postprandial glucose by 3% and 8%, respectively, whereas fasting plasma glucose, insulin requirements, and BMI remained unaltered. There were no major hypoglycemic episodes and none of the participants developed edema (365). In a similar study, pioglitazone (15-45 mg once daily) was given to lean newly diagnosed children and adolescents with T1D (HbA1c 6.7%) for 24 weeks with no effect in any of the parameters investigated (366). The lack of efficacy of pioglitazone likely results from normal body weight and/or young age and short diabetes duration of the participants indicating no relevant insulin resistance. Administration of rosiglitazone (4 mg twice daily) along with insulin to obese adults with T1D (average HbA1c 7.9%, BMI 33 kg/m^2^, diabetes duration 21 years) led to significant reductions in HbA1c, blood pressure, and cholesterol levels, without an increase in insulin requirements (367). Despite these positive metabolic results, the possible side effects of thiazolidinediones (weight gain, bone fractures especially in women, precipitation of congestive heart failure) limit their use in T1D.

### SGLT2 Inhibitors

SGLT2 inhibitors (SGLT2i) increase urinary glucose excretion and natriuresis, improve hyperglycemia, decrease glucotoxicity and oxidative stress, promote weight loss, and reduce systolic blood pressure. Common adverse events include urinary tract/genital infections and diabetic (also euglycemic) ketoacidosis (355). Empagliflozin and dapagliflozin have been widely investigated as adjuncts to insulin in T1D. In the EASE trials (368, 369), administration of empagliflozin (2.5, 10, or 25 mg) along with insulin to overweight people with T1D for 26 to 52 weeks, induced a progressive reduction in HbA1c from 0.28 to 6.4%, body weight from 0.54 to 13.3%, and insulin dose per day from 0.53 to 12.7%. In the DEPICT trials (370), administration of 5 or 10 mg dapagliflozin along with insulin to overweight individuals with T1D for 52 weeks decreased HbA1c by 0.2 and 0.23%, body weight by 2.57 and 3.34 kg, and daily insulin dose by 8.3 and 10.51%, respectively. In a placebo-controlled double-blind cross-over study, short-term administration of dapagliflozin (10 mg for 3 days) did not improve whole-body sensitivity of glucose disposal to insulin during euglycemic-hyperinsulinemia (371). Intervention studies directly assessing insulin sensitivity after a longer-term period of SGLT-2i treatment are however lacking. In all studies, hypoglycemia was rare with similar frequency for empagliflozin, dapagliflozin, and placebo. The incidence of diabetic ketoacidosis was 0.8%, 4.3%, and 3.3% with 2.5, 10, and 25 mg empagliflozin, respectively, and 4% and 3.5% with 5 and 10 mg dapagliflozin, respectively. Canagliflozin has also been investigated in people with T1D for 18 weeks vs placebo. Administration of 100 or 300 mg canagliflozin induced similar reductions of HbA1c >0.4%, while total daily insulin dose was reduced by 4.1 and 7.6 units/day with the 2 doses of the drug, respectively; the decrease was mainly due to basal insulin. Body weight was also reduced by 3.4% and 5.3% for 100 and 300 mg, respectively. The treatment did not increase hypoglycemia risk, while adverse events included diabetic ketoacidosis mainly related to a reduction in insulin dose or illness and insulin resistance (372).

In the TANDEM trials (373, 374), administration of the dual SGLT1/SGLT2 inhibitor sotagliflozin in combination with insulin for 52 weeks reduced HbA1c, body weight, and insulin dosage by 0.36%, 2.92 kg, and 8.2%, respectively; continuous glucose monitoring demonstrated up to 3 hours more time in the range 70 to 180 mg/dL. Low risk of hypoglycemia (3.6%) and diabetic ketoacidosis (1.9%) were demonstrated with sotagliflozin. However, it should be noted that, in contrast to the low incidence of diabetic ketoacidosis in clinical trials, its frequency has been found substantially elevated with these drugs in real-world settings (375). In a recent report with sotagliflozin in T1D (376) the importance of baseline measurements of β-hydroxybutyrate prior to initiation of SGLT2i was proposed to minimize the likelihood of diabetic ketoacidosis during treatment. A real-world study (377) investigated the efficacy, safety, and cardio-renal outcomes of SGLT2i and GLP-1RA in 2.184 individuals with T1D treated with these agents together with insulin for at least 6 months. SGLT2i revealed an HbA1c reduction of 0.2%, preservation of renal function, and lower risk of heart failure and hospitalization for heart failure, confirming the cardio-renal protection of this class of drugs in T1D as in T2D. Taken together, considering their off-label status, mainly due to increased ketoacidosis risk and lack of outcome trials, the widespread use of SGLT2i along with insulin in people with T1D cannot be routinely recommended (378).

### GLP-1 Receptor Agonists

GLP-1 receptor agonists (GLP-1Ras) promote weight loss by central mechanisms, delay gastric emptying/intestinal glucose absorption, inhibit glucagon secretion, facilitate insulin action on the liver, muscle, and endothelium by direct and indirect mechanisms, and exert potent antioxidative and anti-inflammatory effects (355). In T1D, these effects may help to increase tissue sensitivity to insulin and maintain the residual β-cell mass (379). Exogenous insulin treatment and normalization of glycemia in T1D cannot reverse increased glucagon secretion (see “Glucagon, Islet Amyloid Polypeptide”) and, therefore, targeting hyperglucagonemia with GLP-1RAs could be a useful therapeutic intervention (159).

In obese individuals with T1D and insufficient glycemic control, administration of liraglutide as an add-on to insulin for 24 weeks decreased body weight and insulin dose per day by 6.8 kg and 11.2 Units, respectively, while HbA1c was not improved more than in the placebo group. Adverse events included nausea and vomiting which, however, were not severe and subsided soon following dose titration (380). These results were confirmed in the ADJUNCT-ONE/ADJUNCT-TWO placebo-controlled trials in overweight/obese people with T1D. Notably, liraglutide was more effective on HbA1c in participants with higher BMI and residual C-peptide secretion (381, 382). In a retrospective study, treatment of obese people with T1D (average HbA1c 7.6%, BMI 33.5 kg/m^2^) with weekly GLP-1RA semaglutide for a year decreased HbA1c, BMI, and glucose variability (SD and coefficient of variation with continuous glucose monitoring) by 0.66%, 7,9%, and 2.5%, respectively, with no change in daily insulin dose; no episodes of severe ketoacidosis or hypoglycemia were reported (383). Dual GLP-1/GIP receptor agonist tirzepatide was recently proposed to significantly reduce body weight by up to 10.5% at 6 months (384) or 18.5% at 1 year (385) after therapy initiation and improve HbA1c and time in range in humans with overweight/obesity and T1D while being well tolerated. While weight loss, improved glycemic control, and reduction in insulin dose may imply an improvement in insulin sensitivity, studies on GLP-1RA treatment using gold-standard methods to assess insulin sensitivity are still needed in different cohorts with T1D.

In a real-world study of people with T1D treated with GLP-1RAs or SGLT2i for 1 year, both agents induced similar reductions in HbA1c (from 7.7% to 7.3%, and from 7.9% to 7.3%, respectively). However, GLP-1RAs reduced body weight, basal plus prandial dose of insulin/day, and total cholesterol/low-density lipoprotein, whereas SGLT2i reduced only basal and no prandial insulin/day and had no effect on body weight or plasma lipids. There was a 4-fold increase in diabetic ketoacidosis in SGLT-2i vs GLP-1RA users, while the incidence of hypoglycemia was small and identical in both groups (378, 386).

Considering the current issues of high cost and low availability and their off-label status, widespread use of GLP-1RAs and novel coagonists along with insulin in people with T1D cannot be routinely recommended at present but may play a role in future treatment concepts once outcome trials are available (378).

### Pramlintide

Pramlintide is an amylin analog, which mimics amylin action by delaying gastric emptying/intestinal glucose absorption, suppressing postprandial glucagon levels, and increasing satiety. In T1D, the addition of pramlintide to meal-time insulin for 29 weeks reduced postprandial hyperglycemia, body weight, and daily insulin dose by 60%, 1.3 kg, and 28%, respectively (387), and improved oxidative stress (388). Nevertheless, the multiple daily injections required with meals, and the nausea frequently associated with its use have limited its clinical application.

## Conclusions and Future Perspectives

Insulin resistance in muscle, adipose tissue, liver, and vascular endothelium is a prominent feature and an integral part of T1D pathophysiology, develops early during its progression, and is sustained after clinical diagnosis. Contributing factors for insulin resistance in T1D include peripheral insulin administration, weight gain, physical inactivity, and sleep disturbances operating through glucotoxicity, lipotoxicity, hyperinsulinemia, hyperglucagonemia, low-grade inflammation, and impaired mitochondrial function and oxidative stress. Results are sometimes equivocal owing to cohort selection, differences in glycemic control, physical fitness level, and diabetes duration, all playing important roles. Of note, the expected involvement of these risk factors and mechanisms does not always explain the presence of insulin resistance in T1D suggesting a unique phenotype different from T2D. The reciprocal relationship between insulin resistance and impaired endothelial function provides the ground for the development of diabetes-related complications. Concerning the contribution of insulin resistance to chronic complications, it should be noted that the various studies have differently taken confounders into account.

The pathophysiology of T1D is likely multifactorial. In some cases, it may be primarily driven by autoimmunity, whereas in other cases other factors may initiate the destruction of β-cells and hyperglycemia, with autoimmunity to follow. Within this context, the causality in the interplay between insulin resistance and autoimmunity is still unclear.

In clinical practice, individualized physical activity and CSII compared to multiple insulin injections insulin treatment have been shown to improve insulin sensitivity and glycemic control and reduce insulin dose. Several agents improving insulin resistance directly or indirectly, such as metformin, GLP-1RAs, SGLT-2i, and pramlintide have been examined, but are not widely used in routine clinical care at present along with insulin due to limited efficacy, side effects, and a limited number of real-world outcome trials in people with T1D.

Although insulin resistance seems to be an important issue for the clinical care of T1D, its gold-standard assessment is not recommended for clinical routing because the euglycemic–hyperinsulinemic clamp is costly, personnel-intensive, and time-consuming. Nevertheless, the HOMA-IR can serve as a rather simple alternative at least in the preclinical stages and possibly in recent-onset T1D. In preclinical T1D, postprandial insulin sensitivity indexes obtained from OGTT, or mixed meals can also be applied. In overt T1D, eGDR remains the only surrogate index of insulin resistance, which, however, should be interpreted with caution due to limitations (see “Contribution of Individual Tissues to Insulin Resistance”).

Regarding the role of insulin resistance in the pathophysiology and treatment of T1D, several issues remain to be considered by the scientific and clinical community.

1. In individuals genetically susceptible to T1D before autoimmune seroconversion, research is needed with euglycemic–hyperinsulinemic clamps to investigate whether insulin resistance contributes and to what extent to initiating autoimmunity.
2. Although exercise training and increased physical activity improve insulin sensitivity in people with T1D, the fear of hypoglycemia remains a significant barrier to meeting current guidelines. Active research on simple interventions, such as interrupting prolonged sitting with frequent short bouts of light-intensity activities, is urgently needed to encourage sedentary people with T1D who are unwilling to be involved in structured programs to incorporate physical activity into their everyday schedule.
3. Iatrogenic hyperinsulinemia significantly contributes to inducing insulin resistance in T1D. Optimizing subcutaneous insulin treatment to approach physiological insulin supplementation remains an important goal.
4. Real-world studies are necessary on the metabolic benefits and side effects of daily GLP-1/once-weekly GLP-1/dual GLP-1/GIP receptor agonists, and SGLT-2i in T1D, and their protective effects on CVD and chronic kidney disease progression. Measurements of residual C-peptide before treatment initiation with these agents may be useful to reduce the risk of adverse events. Furthermore, studies directly measuring insulin sensitivity with euglycemic–hyperinsulinemic clamps before and after treatment with these agents are needed.

## Abbreviations

BMI: body mass index
CSII: continuous subcutaneous insulin infusion
CVD: cardiovascular disease
eGDR: estimated glucose disposal rate
EGP: endogenous glucose production
GADA: glutamic acid decarboxylase autoantibody
GDS: German Diabetes Study
HDL: high-density lipoprotein
HOMA-IR: Homeostatic Model Assessment for Insulin Resistance
IAPP: islet amyloid polypeptide
IL: interleukin
IMT: intima media thickness
LDL: low-density lipoprotein
MASLD: metabolic dysfunction–associated steatotic liver disease
MDI: multiple daily insulin injection
MRS: magnetic resonance spectroscopy
NEFA: nonesterified fatty acid
OGTT: oral glucose tolerance test
PKC: protein kinase-C
ROS: reactive oxygen species
SAID: severe autoimmune diabetes
T1D: type 1 diabetes
T2D: type 2 diabetes
VAT: visceral adipose tissue
